# A Global Optimizer for Nanoclusters

**DOI:** 10.3389/fchem.2019.00644

**Published:** 2019-09-27

**Authors:** Maya Khatun, Rajat Shubhro Majumdar, Anakuthil Anoop

**Affiliations:** Department of Chemistry, Indian Institute of Technology Kharagpur, Kharagpur, India

**Keywords:** global optimization, PyAR, nanocluster, binary cluster, ternary cluster, nanoalloys, cluster builder

## Abstract

We have developed an algorithm to automatically build the global minimum and other low-energy minima of nanoclusters. This method is implemented in PyAR (https://github.com/anooplab/pyar) program. The global optimization in PyAR involves two parts, generation of several trial geometries and gradient-based local optimization of the trial geometries. While generating the trial geometries, a Tabu list is used for storing the information of the already used trial geometries to avoid using the similar trial geometries. In this recursive algorithm, an *n*-sized cluster is built from the geometries of *n*−1 clusters. The overall procedure automatically generates many unique minimum energy geometries of clusters with size from 2 up to *n* using this evolutionary growth strategy. We have used our strategy on some of the well-studied clusters such as Pd, Pt, Au, and Al homometallic clusters, Ru-Pt and Au-Pt binary clusters, and Ag-Au-Pt ternary cluster. We have analyzed some of the popular parameters to characterize the clusters, such as relative energy, singlet-triplet energy difference, binding energy, second-order energy difference, and mixing energy, and compared with the reported properties.

## 1. Introduction

A major focus in modern nanoscience is to understand the properties of materials on the atomic scale (Eberhardt, 2002). Subnanometer scale metal clusters are of great interest due to their structural and electronic properties (Baletto and Ferrando, 2005), which makes them useful for applications in various field like nanotechnology, electronics, medical device and catalysis (Saha et al., 2012). The atomic clusters may comprise of atoms of the same element such as in fullerenes or atoms of different elements as in nanoalloys (Johnston, 2002). A molecular-level understanding of small nanoclusters would provide insights into the largely empirical field of nanoscience.

Theoretical study of nanoclusters can help us to understand the smooth transition from atoms to bulk materials, especially the size-dependent evolution of the properties (Jortner, 1992; Edwards et al., 1998). The primary input for the theoretical study is their geometry. While determining the geometry of nanoclusters by experiments is extremely difficult, the atomic structure of clusters can be predicted theoretically by geometry optimization tools that are specifically designed for global optimization (Zhao et al., 2017).

Global optimization of functions is an essential part of various research fields and have many real-life applications (Floudas and Gounaris, 2008; Barbati et al., 2012; Khare and Rangnekar, 2013). The global optimization (GO) is the process of finding the best solution, “global maximum” or “global minimum” (GM), based on one or more criteria for a mathematically formulated function (Jäger et al., 2018). The global optimization in our context refers to finding the most stable geometry for a particular cluster, that is the lowest energy atomic arrangements on the potential energy surface (PES). The global minima of atomic clusters (Davis et al., 2015; Shayeghi et al., 2015a) are essential as these are often the most likely structure to be formed in the experiment. Thus, finding the global minimum and other low-lying minima on the PES is helpful to interpret the experimental results (Shayeghi et al., 2014, 2015b; Götz et al., 2016).

The efficiency of geometry optimization (GO) algorithm is crucial for the success in the attempts to understand the cluster science. Some of the popular GO algorithms are Genetic Algorithms (GA) (Johnston, 2003), Basin Hopping (BH) (Wales and Scheraga, 1999), Particle Swarm Optimization (PSO) (Lv et al., 2012; Shi et al., 2019), Artificial Bee Colony (ABC) (Zhang and Dolg, 2015), Simulated Annealing (Kirkpatrick et al., 1983), Threshold Algorithms (Schön et al., 1996) etc. These general GO algorithms are employed in the studies of metal clusters with varying degrees of success. As for any applications of GO, there is no universal method that works for all the molecular systems in chemistry and is an open area of research.

A major challenge in any GO method is the computational complexity, the exponential increase in the search space with system size (Doye and Wales, 1998). A GO algorithm must combine a locally confined search with the wide exploration of the regions without revisiting the same regions (Heiles and Johnston, 2013) in the PES in a computationally effective way. The fine balance of local search and global exploration is required. The re-examination of a minimum only gives redundant information wasting computational resources. On the other hand, confining the search only to a small neighboring area does not allow the algorithm to find the GM in other funnels on the PES. Metadynamics algorithms overcome the revisiting problem by adding time-dependent repulsive bias potential function of collective variables to discourage revisiting the already visited areas. Tabu-search based algorithms (Glover, 1986, 1989, 1990) store the information of previously visited areas to avoid the searching of the already explored region.

In this article, we explain our strategy to find the global minima geometries of atomic clusters—unary, binary and ternary nanoclusters. We have combined two strategies to improve the efficiency: the Tabu-search algorithm to reduce the time spent on the already found minima and a novel recursive approach to reduce the search space by making use of the solutions from the smaller problem. That is, we build the solutions of *n* sized cluster based on the solutions of *n* − 1 sized cluster. This way, the unique geometries of cluster size *n* can be built bottoms-up starting from the single atom. This method is particularly useful for studying the evolution of structure and properties with the growth of cluster size. We have discussed the implementation and the validation by applying on the known metallic clusters. We have compared the geometries and a few representative properties of the clusters generated by our algorithm with the reported geometries and corresponding properties.

## 2. Theoretical Approach

### 2.1. Cluster Building and Optimization

**Figure 1:**
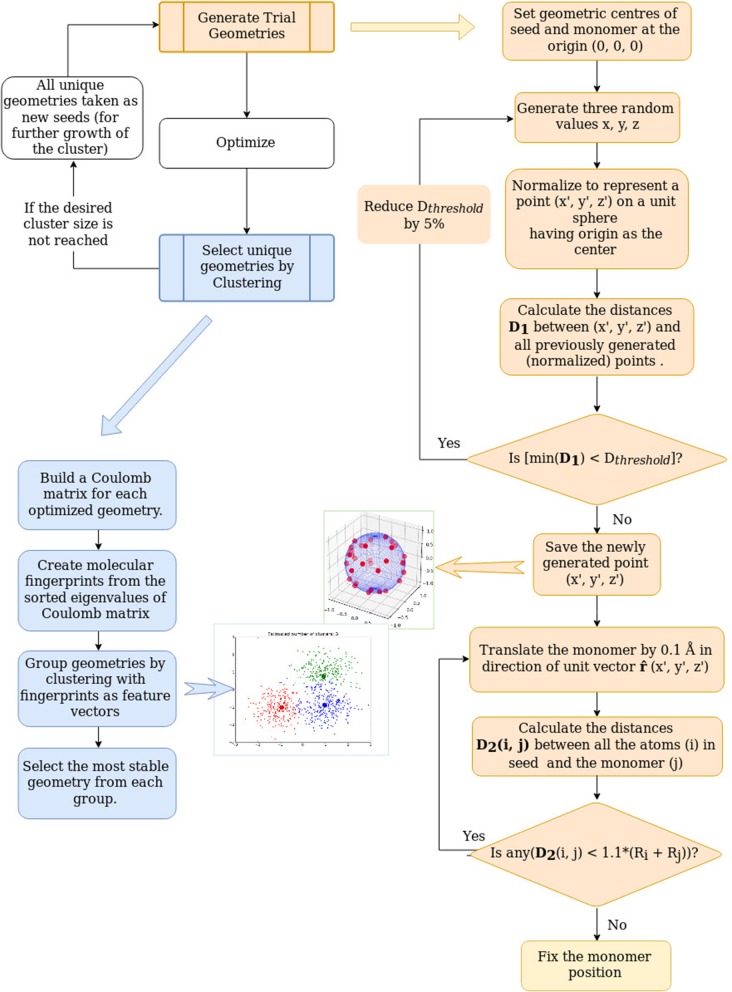
The flowchart for the cluster building method.

Our method for the global optimization of the geometries of atomic clusters is an adaptation of our approach for the automated exploration of reaction and aggregation implemented in PyAR (Nandi et al., 2017; Anoop, 2019) program. In this section, we will explain the philosophy and implementation of the aggregator modules used for the building of nanoclusters (Figure 1). The global optimization for nanoclusters in PyAR involves two parts, generation of several trial geometries and gradient-based local optimization of these trial geometries. In our algorithm, the search for solutions of *n*-sized cluster make use of the solutions from the search on the *n* − 1 sized clusters. At each cycle, the problem is reduced to find the best relative orientations between two species. This approach is analogous to finding the solution of the traveling salesman problem with N cities by adding one more city to the solution of the problem with N-1 cities. The overall procedure automatically generates several unique minimum energy geometries of clusters with size up to *n* using our evolutionary growth strategy.

This process can be imagined as growing the cluster by adding atoms one by one. The method is similar to the cluster-fusion algorithm of Solov'yov et al. (2004). When the second atom is added to the first one, there is only one possible geometry and there is only one variable—the distance between the atoms. The trial geometry for the dimer is generated as follows. The first atom (called as seed) is placed at the origin of the Cartesian coordinate system. For placing the second atom (named monomer), the value for the *x*-coordinate is generated as a random number between 0 and 1. Then, the value of *x* is increased in small steps of 0.1 Å until *x* is larger than the sum of covalent radii of both atoms (*x* > (*R*_*a*_ + *R*_*b*_)). This way, the second atom is placed in the *X*-axis at a distance of no close-contact between the atoms.

The third atom could be placed anywhere in the *xy*-plane at a distance from the existing atoms of the dimer avoiding close-contacts. Here, the dimer is the seed, and the atom is the monomer. The *xy*-plane (the search space) is divided into four quadrants. The new atoms are placed in each quadrant sequentially. The quadrant is chosen by generating random numbers for *x* and *y* coordinates within a suitable range to fit a particular quadrant. The new coordinates created by these random numbers are normalized so that the point is at a unit distance from the origin. As described above, the third atom initially placed at this position is translated away from the origin to avoid any close contacts.

The search space for the addition of the fourth atom and further on is three-dimensional. The 3D space around the trimer (and larger *n*'mers) is divided into eight octants. The new atoms are placed in random positions at unit distance from the origin in each of these octants sequentially. The reason for dividing the space into octants is to distribute the new trial geometries evenly so that even with a few trial geometries, there is a chance of exploring different region of space and getting dissimilar geometries. This way, *N* trial geometries are generated. *N* is a user-provided parameter. All the trial geometries will be optimized using local, gradient-based optimizers. The optimizations are done by the interfaced software as described later in this section.

Some of the optimized geometries obtained by the gradient-based optimization of these trial geometries may belong to the same minima in the PES, with small differences in geometrical parameters depending on the convergence criteria. Comparison of geometries based on Cartesian coordinates such as RMSD of the atomic positions may fail because the optimization may reorient the molecule, and the Cartesian coordinates are not rotationally invariant. Besides, the same geometry with different ordering of atoms will also be shown as different geometries by such comparisons. Therefore, we have implemented various molecular representations to find the similarity.

One of such representations that we have used in this work is the molecular fingerprints, computed as follows. An n-by-n matrix, known as Coulomb matrix (Rupp et al., 2012; Sadeghi et al., 2013), is made in which the off-diagonal elements are the pairwise Coulomb repulsions $\frac{Z_{i}Z_{j}}{R_{ij}}$, and the diagonal elements are $Z_{i}^{2.4}/2$. The *Z*_*i*_ and *Z*_*j*_ are the core charge of atom i and j. The Coulomb matrix is diagonalized. The sorted eigenvalues are considered as the molecular fingerprints. The fingerprint is used as the feature vector for clustering algorithm (see below) and the euclidean distance between the fingerprints is used as the measure of similarity.

Using the molecular fingerprint representation, these optimized geometries are analyzed and clustered into groups (up to 8 clusters) of similar geometries using clustering algorithms (Nandi et al., 2018) in Scikit-learn (Pedregosa et al., 2011) python library. The most stable geometry from each of these clusters are selected as the minima for this *n*'mer and the most stable among the minima is the global minimum geometry for this *n*'mer. All of these minima are considered for further growth by adding a new atom. This way the degree of freedom of *n*'mer (3*N* − 6) is reduced to 3.

Besides the reduction in complexity, the other significant improvement to increase the efficiency is to avoid revisiting the already visited regions. In our context, we store all the randomly created points and compare the new point with the stored points. For a reasonable comparison, all the positions are generated at a unit distance from the origin, i. e. positions lie on the surface of the sphere of a unit radius (1 Å). If the new position is within the threshold distance from any of the stored positions, the new position is rejected. This threshold distance is initially set as 0.3Å and is increased by 5 % in each cycle. As this idea is adapted from Tabu-search algorithm (Glover, 1986, 1989, 1990), the list of stored positions is referred as the Tabu list. This method of filtering the position makes sure that the trial geometries are sufficiently dissimilar.

The *N* trial geometries created by the method explained above will be optimized with the electronic structure programs that are interfaced with PyAR. Currently we have interfaced with Gaussian 09/16 (Frisch et al., 2016), MOPAC (Stewart, 2016), PSI4 (Turney et al., 2012), ORCA (Neese, 2018), Turbomole (Furche et al., 2014), XTB (Grimme et al., 2017). The user can choose the program and the methods (functional-basis set, semiempirical method). There are few rounds of optimizations. The full set of trial geometries will be initially optimized by loose convergence setting. After filtering similar geometries based on the similarity based on molecular fingerprints, a smaller set of selected geometries will be optimized with standard convergence criteria. In principle, we can also make the automatic procedure to use initial screening with fast and less accurate methods followed by calculations with slow and more accurate methods on a smaller number of geometries.

The methodology described above is for the homometallic clusters. We have extended the procedure to create the binary, ternary and other heteroatomic clusters that are even more interesting and challenging. For making binary clusters, we use both the input atoms as the seed and the monomer instead of one being the seed and the other as the monomer. The procedure, implemented as binary_aggregator, generates all combinations of binary clusters of size ranging from A_1_B_1_ to A_m_B_n_. The algorithm first treats “A” as the seed and “B” as the monomer and repeats the cycle until the number of “B” atoms reaches *n*. Hence, the row of the matrix is built ranging from A_1_B_1_ till A_1_B_n_. When B is considered as seed and A as the monomer, another row is built ranging from A_1_B_1_ till A_m_B_1_. Similarly, by using A_x_B_y_, x < m and y < n, other rows of the matrix can be generated.

We added another layer over the binary_aggregator to build the ternary clusters by including a third element. The ternary_aggregator operates analogously by adding the element C sequentially to each combination of binary clusters made by binary_aggregator. The new monomer is added until it reaches its desired size of the third element. Thus, for each of the binary cluster (A_i_B_j_; i = 1-m, j=1-n), the 3rd element is added as a monomer to generate ternary clusters ranging from (A_m_B_n_C_1_) to (A_m_B_n_C_l_) where l is the maximum number of element C.

Current procedures for binary and ternary clusters are expensive because we used exhaustive enumeration. Exhaustive exploration is required until we find some guiding principles for understanding the mixing behaviors of these alloys.

### 2.2. Properties of Clusters

The relative stabilities of the clusters built using the above described methods can be calculated using the following popular parameters.

#### 2.2.1. Homometallic Clusters

##### 2.2.1.1. Relative energy (RE/eV):

The energy of a cluster compared with the most stable isomer (GM). The higher RE means a lower stability.

##### 2.2.1.2. Singlet triplet energy difference (Δ*E*_*ST*_/eV):

The energy difference between the singlet and triplet state is Δ*E*_*ST*_ = *E*_*triplet*_ − *E*_*singlet*_. The cluster with a positive Δ*E*_*ST*_ has a singlet ground state, and the cluster with a negative Δ*E*_*ST*_ has a triplet ground state.

##### 2.2.1.3. Binding energy per atom (BE/eV):

The binding energy per atom (BE or BEPA) is calculated by Equation (1):

(1) $$\begin{array}{r} BE=\frac{1}{N}\left[E_{n}-nE_{1}\right] \end{array}$$

where, *E*_*n*_ is energy of n atomic cluster; *n* is the cluster size or aggregation number; *E*_1_ is the energy of an atom.

##### 2.2.1.4. Second-order energy difference (δ^2^*E*(*n*), SOD/eV):

The SOD indicates the higher stability of a cluster of *N* atoms relative to its heavier and lighter neighbors. Therefore, δ^2^*E*(*n*) is more relevant in interpreting experimental mass spectral intensities than the BE (Rogan et al., 2005). Large maxima of δ^2^*E*(*n*) shows the higher probability of finding these clusters.

(2) $$\begin{array}{r} \delta^{2}E\left(n\right)=E_{n+1}+E_{n-1}-2E_{n} \end{array}$$

where, *E*_*n*+1_ is the total energy of *n* + 1 atomic cluster; *E*_*n*−1_ is the total energy of *n* − 1 atomic cluster; *E*_*n*_ is the total energy of *n* atomic cluster; and *n* is the cluster size.

#### 2.2.2. Energy Parameters for Binary and Ternary Nanoalloys

##### 2.2.2.1. Binding energy per atom (BE/eV):

The BE for binary and ternary clusters (Song et al., 2005; Demiroglu et al., 2017) is given by Equations (3) and (4):

(3) $$\;E_{b}\;=\frac{1}{N}[E_{tot}(A_{m}B_{n})-mE_{tot}(A_{1})-nE_{tot}(B_{1})]$$

(4) $$\;E_{b}\;=\frac{1}{N}[E_{tot}(A_{m}B_{n}C_{l})-mE_{tot}(A_{1})-nE_{tot}(B_{1})-lE_{tot}(C_{1})]$$

where, *m*, *n*, and *l* are the numbers of A, B, and C atoms; *E*_*tot*_(*A*_1_), *E*_*tot*_(*B*_1_), and *E*_*tot*_(*C*_1_) are the electronic energies of a single A, B or C atom and *N* is the total number of atoms (*N* = *m* + *n* + *l*) in the particular cluster.

##### 2.2.2.2. Mixing energy (ME/eV):

The mixing energy (Song et al., 2005; Pacheco-Contreras et al., 2018) is an indicator of the stability of the binary cluster with respect to its unary counterpart, given by Equation (5):

(5) $$\;\delta =E_{tot}(A_{m}B_{n})-m\frac{E_{tot}(A_{m+n})}{m+n}-n\frac{E_{tot}(B_{m+n})}{m+n}$$

where, *E*_*tot*_(*A*_*m*_*B*_*n*_) is the total energy of the alloy, *E*_*tot*_(*A*_*m*+*n*_) and *E*_*tot*_(*B*_*m*+*n*_) are the total energies of the pure metal clusters, A and B of the same size (*m* + *n*). A negative value of δ means a decrease of energy upon mixing and therefore, a favorable mixing.

## 3. Computational Details

We used the PyAR program to build the clusters, primarily with the Tight-Binding semi-empirical method, GFN-xTB, with the XTB program (Grimme et al., 2017). This combination is denoted as PyAR|XTB. In a few cases, the selected geometries from PyAR|XTB were reoptimized using PBE0 (Adamo and Barone, 1999) functional and def2-TZVP basis set with the ORCA4.0.1.2 (Neese, 2018) program. These minima from PBE0/def2-TZVP was characterized as true minima with no imaginary frequency. This combined method is denoted as PyAR|XTB||PBE0. We have used another combination where the clusters are built using the ORCA program as the interface using the PBE functional or the BP86 (Perdew, 1986; Becke, 1988) functional and the def2-SVP basis set (Weigend and Ahlrichs, 2005), denoted as PyAR|ORCA. We have added Grimme's dispersion corrections (D3-BJ) (Grimme et al., 2011) in all DFT calculations. We have used effective core potential (ECP) (Pettersson et al., 1983) in the DFT calculations to add the relativistic effect for all the transition metals.

## 4. Results and Discussion

We have built various metal clusters—homometallic nanoclusters, bimetallic and trimetallic nanoalloys. In this work, our focus was to validate our approach for its ability to generate the global minimum (GM) and other unique local minima and reproduce the qualitative trends in various properties. Therefore, we have chosen the clusters and alloys that are studied extensively—Pd, Au, Pt, and Al homometallic clusters and Ru-Pt, Au-Pt, Ag-Au-Pt nanoalloys. We have compared the GM geometries and few other low-lying local minima with the corresponding reported geometries. We calculated few properties such as relative energy, binding energy, singlet-triplet energy difference, second-order energy difference, and mixing energy of the clusters and alloys made by our program and compared with the values and trends reported in the literature. Due to the difference in electronic structure theories in different studies, differences are expected in absolute numbers, but overall trends were similar.

### 4.1. Homometallic Nanoclusters

#### 4.1.1. Palladium

**Figure 2:**
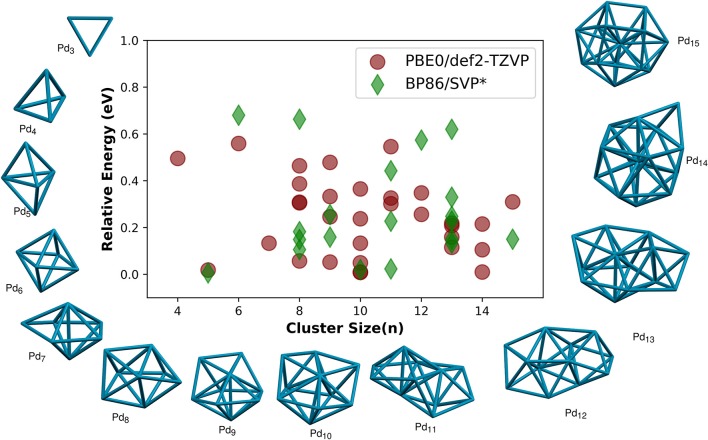
Relative energies (RE/eV), the energy of the optimized isomer compared with the energy of their respective global minimum isomer, of palladium clusters of size (*n* = 4 − 15). The corresponding RE reported using BP86/SVP (Nava et al., 2003) is also plotted for comparison. Global minimum geometries of size *n* = 3 − 15 atoms are shown. The geometries are obtained by using PyAR|XTB calculation followed by optimization at PBE0/def2-TZVP.

The first example for this study of nanoclusters is the palladium nanoclusters. We have located the unique geometries of Pd_n_ (*n*=2–15) clusters using our algorithm implemented in PyAR program. We used two different methods for the global optimization, PyAR|XTB and PyAR|ORCA(PBE). We have also used a two-layer approach in which the search for geometries is done by one method and the selected geometries are optimized again at a different method. For example in the method named as PyAR|XTB||PBE0, the search was done with PyAR|XTB and the geometries selected by this method were further optimized with PBE0. We have employed two more DFT functionals in this study, PyAR|XTB||B3LYP and PyAR|XTB||M06. We have further compared the geometries of Pd_n_ clusters in singlet and triplet electronic states. The global minimum geometries of singlet Pd_n_ clusters are shown in Figure 2.

Only one minimum was found for triatomic palladium clusters, Pd_3_, which has a triangular geometry. The shape of Pd_3_ is slightly distorted from the equilateral triangle with the base angle of 59.9°; such non-equilateral geometry was also reported by Nava et al. (2003). The average bond length and bond dissociation energy are 2.54 Å and 2.57 eV at PBE0/def2-TZVP compared to the values from CAS/MRSDCI level calculation (Balasubramanian, 1989) which are 2.67 Å and 3.28 eV.

As the cluster size grew, the program has selected more than one unique structures for clusters with *n* = 4–15. The relative energies (RE, the energy compared to the global minimum isomer) of all the non-global-minimum geometries are shown in Figure 2, along with the results from Nava et al. (2003) for comparison. All the larger Pd_n_ clusters, *n* > 3, have three-dimensional global minima. Some of these GM geometries are discussed below.

The most stable structure for Pd_4_ cluster is tetrahedral. Bond dissociation energy is 4.77 eV at PBE0/def2-TZVP level compared to 5.07eV at the MRSDCI level calculations (Dai and Balasubramanian, 1995). The bond length is 2.62 Å at PBE0/def2-TZVP, 2.68 Å at MRSDCI (Dai and Balasubramanian, 1995) and 2.61 Å using other DFT calculations (Xiao et al., 1999). We found another minimum, a bicyclic, non-planar, *butterfly*-like geometry, not reported before, which is 0.50 eV higher in energy than the tetrahedral GM structure. Global minimum geometry of Pd_5_ is trigonal bipyramid. The average bond length in this geometry is 2.74 Å, and the binding energy of the *TBP* structure we calculated at PBE0/def2-TZVP is 1.34 eV, similar to the reported values from the DFT calculation (Wen et al., 2018) using GGA functional (BP/DNP), 2.704 Å and 1.73 eV, respectively. The Pd_6_ cluster has an octahedral global minimum. Thus, the most symmetric platonic geometries–trigonal, tetrahedral, trigonal bipyramidal, and octahedral–are the global minima for Pd_3_-Pd_6_.

The most stable geometry of Pd_7_ from the PyAR|XTB calculations is pentagonal bipyramidal (PBP), but is a non-platonic geometry, octahedral core with one cap when PBE and PBE0 methods were used. The PBP was not a minimum, and the trigonal bipyramid with two caps is the next higher energy isomer that has a RE of 0.13 eV compared to GM in PBE0. In the triplet state, the PBP is the most stable structure at BP86 (Nava et al., 2003) and BLYP (Rogan et al., 2005) levels. According to Nava et al. (2003), the mono-capped octahedral and bicapped-TBP Pd_7_ are only 0.03eV and 0.05eV higher in energy, respectively, compared to the most stable PBP.

The symmetric dodecahedral geometry was found to be the lowest energy cluster for Pd_8_. From Pd_8_ to Pd_13_, pentagonal bipyramidal (PBP) based structures dominate the global minima. For Pd_13_, the most symmetrical icosahedral structure is not the GM in our calculation (R.E. = 0.21 eV), in agreement with the calculations by Nava et al. (2003) and Reveles et al. (2012) in which the symmetric geometry is higher in energy compared to the most stable geometry by 0.13 eV (BP86/SVP) and 0.16 eV (PBE/DZVP), respectively. The Pd_14_ has an icosahedral core with one cap.

We have calculated the selected geometries in the triplet state as the report (Nava et al., 2003) suggested that many of the Pd clusters have triplet ground states. The Δ*E*_*ST*_ is shown in Figure S1. In PBE0, all Pd_n_ clusters have negative Δ*E*_*ST*_, i. e. have triplet ground state, except for Pd atom. The ground state of the Pd atom has a closed-shell electronic configuration. The dimer is well established as a triplet ground state in the literature (Lin et al., 1969; Zacarias et al., 1999; Nava et al., 2003), which is reproduced by our DFT result as well—the singlet Pd_2_ has higher energy (0.45 eV) than its triplet state. The dissociation energy of dimer is 0.64 eV which is in agreement with the experimental dissociation energy 0.73 ± 0.26 eV (Lin et al., 1969) as well as various density functional calculations done by Zacarias et al. (1999). The GFN-xTB results, however, showed that all the Pd_n_ clusters, except Pd_6_, have singlet ground state. The Δ*E*_*ST*_ in GFN-xTB is large positive for *n* = 1, 3, and 5, but are slightly positive for *n* = 2, 4, 7–15. Thus, Δ*E*_*ST*_ is not well represented by GFN-xTB in this Pd_n_ clusters.

**Figure 3:**
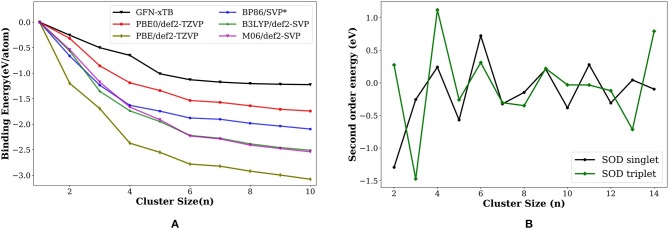
**(A)** Variation of binding energy (BE; eV/atom) with the cluster size for the most stable palladium cluster obtained with different methods. ^*^ values from Nava et al. (2003). **(B)** Second order energy difference (eV) plotted as a function of cluster size (*n*) for the lowest-energy isomers of singlet and triplet state. The geometries obtained using PyAR|XTB calculation were re-optimized at PBE0/def2-TZVP.

The binding energy per atom (BE/eV) increases as the cluster grows, the trend consistently reproduced by all methods (Figure 3A), GFN-xTB, BP86 (Nava et al., 2003), PBE, PBE0, B3LYP, and M06 calculations. The most stable geometries as well as the qualitative features in the overall binding energies gives us a promising strategy for the building of large scale clusters. We can use a two-stage approach where a semiempirical calculations is used for the exploration of minima using PyAR, followed by the optimization in DFT for the selected geometries.

The second order energy difference (SOD; Figure 3B) is useful for understanding the stability of cluster with size *n* compared to the clusters with size *n* − 1 and *n* + 1. The computed SOD for Pd_n_ cluster shows that Pd_2_, Pd_4_, Pd_6_ are more stable than its neighbors. The clusters with even number of atoms are relatively more stable than the ones with odd number of atoms. This observation is in agreement with Rogan et al. (2005) and Wen et al. (2018) which showed that Pd_2_, Pd_4_, and Pd_6_ are relatively stable than their neighbors.

In short, the study of Pd_n_ clusters show that the GM structures obtained by our methodology are in good agreement with those from the reported GM structures by other studies (Nava et al., 2003; Rogan et al., 2005). We have studied three more homometallic clusters, Au, Pt and Al, and we have focused different aspects of each clusters below.

#### 4.1.2. Gold

After the study of Pd nanoclusters, we have applied our method to explore the minima of gold clusters using PyAR|XTB(GFN-xTB) and PyAR|ORCA(BP86/def2-SVP). We have generated geometries up to n = 10 with PyAR|DFT and up to 20 with PyAR|GNF-xTB. The GM structures for *n* = 4 − 8 obtained from our calculations in both the methods are identical with reported structures from CCSD(T) calculations Shi et al. (2010); Baek et al. (2017). Au_4_ obtained as a rhombus type structure. The global minimum of gold pentamer is W-shaped, and the hexamer is a planar triangle. The GM of Au_7_ has an Au capped the edge of planar triangular Au_6_. The Au_8_ has GM where an Au is capped to each edges of a square.

For Au_3_, the PyAR|BP86 run found triangular and bent geometries. While, the global minima at CCSD(T) level is triangular (Baek et al., 2017), our results at BP86, PBE and PBE0 shows the bent geometry as GM. The bent structure was not a minima with PyAR|GFN-xTB and M06 functional, the optimization resulted in a triangular geometry. Thus, other than Au_3_, all the other geometries for Au_n_; *n* = 4 − 8 have identical geometries in GFn-xTB and DFT.

The bond length of gold dimer is calculated as 2.472 Å by GFN-xTB and 2.543 Å by BP86 which are in good agreement with the experimental value 2.490 Å. One of the important energy parameters, cohesive energy (CE) of Au_2_ is 1.117 eV by our BP86 calculation. This is comparable with 1.1481 eV at the CCSD(T) level (Shi et al., 2010) and 1.1524 eV from experiment (Bishea and Morse, 1991). The CE by GFN-xTB, 4.005 eV, is too high. For the gold trimer, the calculated CE is 1.172 eV, the reported results are 1.161 eV (Shi et al., 2010) and 1.255 eV (James et al., 1994). Au_4_ has a CE of 1.487 eV, comparable with the CCSD(T) value of 1.556 eV (Shi et al., 2010). While the results from our BP86 calculations follow the trend with the reported CCSD(T) (Shi et al., 2010) and experimental (Bishea and Morse, 1991; James et al., 1994) results, the GFN-xTB overestimates the CE.

**Figure 4:**
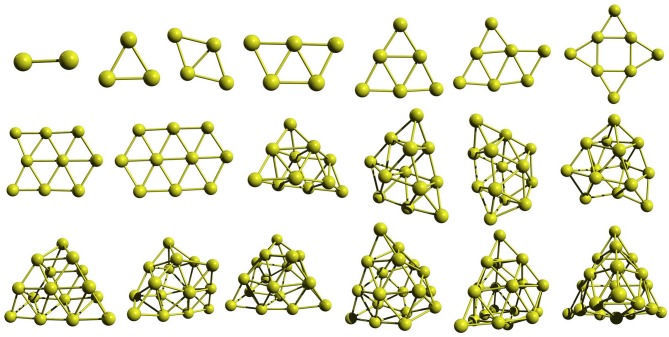
The global minimum structures of Au_n_; *n* = 2–20, obtained by the global search using PyAR|XTB.

The GM geometries shown in Figure 4 reveal that the gold clusters have flat GM up to the cluster size of ten atoms. Al_11_ has a 3D geometry. Thus, our approach is able to capture the structure evolution from 2D geometry to 3D geometry that can be attributed to the use of multiple unique seed geometries to build the clusters rather than using only the GM geometry. All the selected geometries of Au_10_ and Au_20_ is shown in Figure S2. The lowest-energy isomers of Au_10_ below 0.4 eV include planar and 3D geometries—the best two are planar. As we have seen above, while the Pd clusters prefer 3D geometries throughout the size range we have studied, the gold clusters remain flat for small sizes, up to 10 in GFN-xTB and BP86 levels.

To study the effect of the number of orientations (**N**) used in the run, we carried out separate runs with different values of **N**. As the size of the cluster increases, the **N** becomes more and more important. For example, the GM (shown with ^**^ in Figure S2A produced by one of the PyAR|XTB run with ***N*** = 8 is only one of the local minima, not a GM, in the GA-DFT study (Shayeghi et al., 2015a). However, another run with more orientations along with GFN-xTB resulted in the GM from the GA-DFT and other calculations (Gotz et al., 2013; Shayeghi et al., 2015a). Similar run with DFT also produced the latter GM. The effect is more evident in the Au_20_ cluster.

The Au_20_ has a highly symmetric tetrahedral (T_d_) geometry which is one of the most often found structures in the experiment (Gruene et al., 2008) and is one of the most stable geometry in various theoretical calculations (Assadollahzadeh and Schwerdtfeger, 2009; Shayeghi et al., 2015a). The lowest-energy Au_20_ isomers in the range below 0.5 eV are shown in Figure S2B). Our geometries are comparable to the ones from previously studied GA-DFT, BH-DFT calculations and the experimental result (Gruene et al., 2008; Shayeghi et al., 2015a; Zhao et al., 2017).

**Figure 5:**
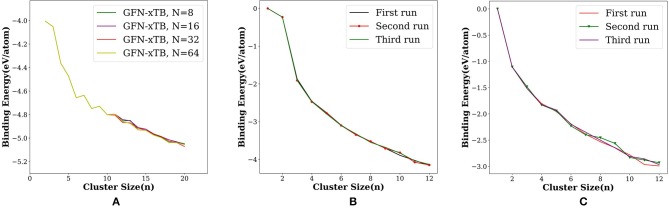
Binding energy per atom (eV/atom) for **(A)** Au_n_; *n* = 2–20 with the number of orientations (*N* = 8, 16, 32, and 64) and for three different runs done on **(B)** platinum and **(C)** aluminum using PyAR|XTB(GFN-xTB) calculation.

The search for global minimum using only eight orientations was able to locate the tetrahedral global minimum geometry of Au_20_, however, not always. By varying the number of orientations in the search—***N*** = 8, 16, 32, and 64—we checked the probability of getting the global minimum. When the orientation number is 32, GM structure was found in a single run. As one can anticipate, the possible ways in which the new atom can be added to the (*n* −1) ^*th*^ cluster increases on increasing the cluster size. Therefore, we have to increase the number of orientation With increasing cluster size. We have illustrated this by plotting the binding energy per atom for the runs with number of orientation as 8, 16, 32, and 64 (Figure 5A). We have made an option auto for the number of orientation *N* in which *N* doubles after each cycle starting with eight in the first cycle, then *N* increases as 16, 32, 64, 128, 256 and up to a maximum of 512.

#### 4.1.3. Platinum

We studied platinum nanoclusters as the next example as the Pt-based nanoclusters are useful materials with applications in various heterogeneous catalysis. Jennings and coworkers performed GA-DFT searches on small-sized (Pt_n_, *n* = 3–6) platinum clusters to find their GM structures. The study showed that Pt clusters have non-singlet ground states, and the geometry of GM's can vary for different spin multiplicity (Jennings and Johnston, 2013). Thus, we have performed three different global minimum searches with multiplicities 1, 3, and 5 on Pt_n_; n = 3–6 with PyAR|XTB.

**Figure 6:**
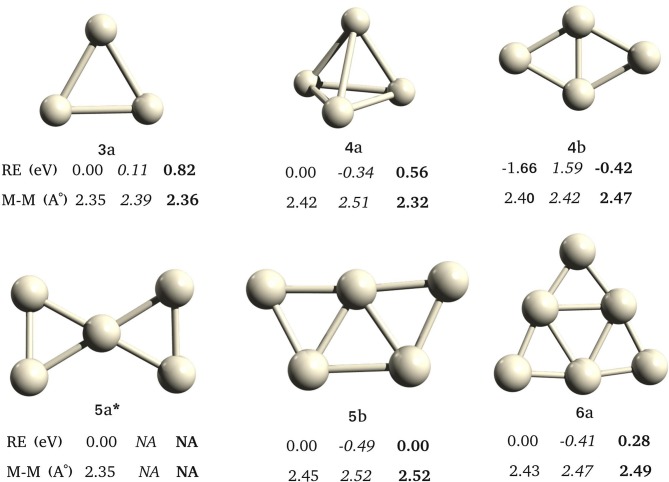
Low energy structures found for pure Pt clusters, from Pt_3_ to Pt_6_, with different spin multiplicities. ^*^Only singlet state was converged for **5a**. Relative energies (RE/ev) and average bond lengths (Å) of singlet, *triplet*, and **quintet** states shown in normal, italics, and bold fonts.

We have observed different global minima for different multiplicities (Figure 6) for Pt_4_ and Pt_5_, in agreement with the GA-DFT study. The Pt_3_ has the same triangular geometry in singlet and triplet states, and singlet is the ground state. There are two geometries, **4a** and **4b** for Pt_4_. The **4b** has the lowest energy in its singlet state. The ground state of **4a** is a triplet, however, it is higher in energy than the singlet-**4b**. The **5a** is minima only in singlet state. The GM for Pt_5_ is **5b** in triplet state. The Pt_6_ has a triplet ground state (**5b**).

#### 4.1.4. Aluminum

The last example for the homometallic cluster in this article is the aluminum cluster. We have built the global minimum structures of Al nanoclusters up to Al_12_ with PyAR|XTB, and up to Al_8_ with PyAR|BP86. There are several theoretical studies on Al clusters at various levels of theory, such as Sutton-Chen empirical potential (Joswig and Springborg, 2003), DFT (Ahlrichs and Elliott, 1999; Rao and Jena, 1999), and CCSD(T) (Shinde and Shukla, 2014; López-Estrada and Orgaz, 2015).

The most stable structure for the trimer, Al_3_ is triangular in both the calculations, PyAR|(XTB, BP86). The bent and linear isomers are 0.53 eV and 0.63 eV higher in energy compared to the most stable structure at BP86/def2-SVP level. We found a planar rhombus geometry for Al_4_ with PyAR|BP86 in agreement with the reported *ab initio* methods. PyAR|GFN-xTB calculation gave a slightly different non-planar rhombus geometry as the most stable structure, but tetrahedron is a minima at GA-Sutton Chen potential. The GM of Al_5_ is a planar W-shaped structure in our calculation (PyAR|(XTB, BP86)) in agreement with the reported minima from *ab initio* calculations.

**Figure 7:**
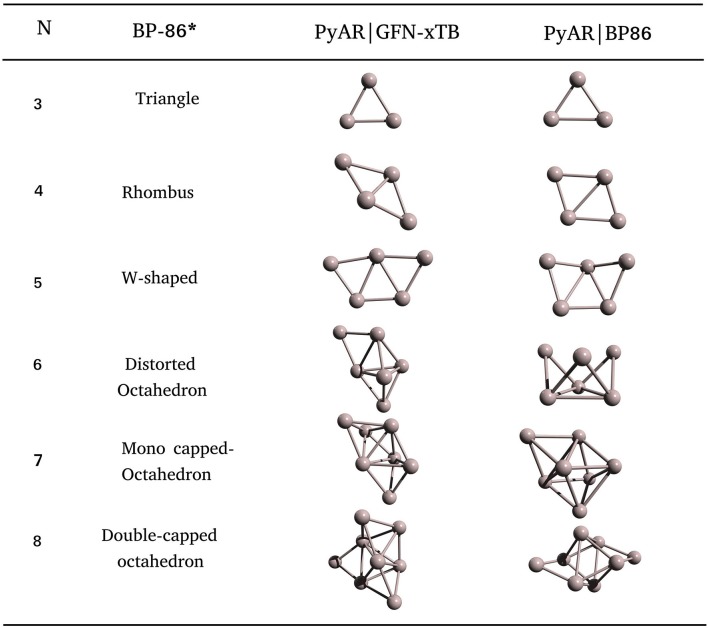
Global minimum structures of Al_n_; *n* = 3–8, obtained by the global search using PyAR|XTB, PyAR|BP86, and reported results (Ahlrichs and Elliott, 1999).

The GM of Al_6_ by PyAR|XTB is a TBP with an edge-cap, but PyAR|BP86 calculation gave a crown-shaped structure as GM (Figure 7). The structure of Al_6_ reported by Jones and Ahlrichs (Jones, 1993; Ahlrichs and Elliott, 1999) is a distorted octahedron, not in agreement with any of our minima. For Al_7_, the trigonal bipyramid with two capped atoms is the GM at PyAR|XTB, while PyAR|BP86 produced a mono-capped octahedron that matched with the reported minima. The octamer Al_8_ showed capped trigonal bipyramid as the minima by PyAR|XTB, octahedral core with two edge-capped by PyAR|BP86 that matched with SC potential (Joswig and Springborg, 2003) and DFT studies (Jones, 1993; Ahlrichs and Elliott, 1999).

#### 4.1.5. General Features

We have studied various features of the approach to finding the global minima of metal nanoclusters. In gold and aluminum clusters, we have compared different methods. All the methods, including semiempirical, produced the same global minima for gold clusters, while the GM was highly dependent on the method for Al clusters. Hence, the choice of appropriate method is crucial.

Some of the clusters have different structural motifs for different sizes. Our method was able to capture the changes in the structural motifs. The global minima for gold clusters were flat upto the size of ten and were 3D geometries afterwards. In order to check these structural changes, we have carried out PyAR|XTB calculation on carbon clusters. We have observed minima corresponding to linear, monocyclic, tricyclic, and the bowl shapes (Figure S3).

We have checked the variation in binding energy per atom on varying the number of orientations (**N**) in the Au cluster (Figure 5A). The use of more orientations was crucial, especially for the larger clusters. We have then checked the variation in BE for three separate runs for Pt and Al clusters. While the plot of BE for each run (Figure 5B) shows nearly perfect overlapping lines for Pt clusters, the BE's sightly differ for Al clusters (Figure 5C). As the cluster size increases, the search space increases. Hence, either increase the **N**, or carry out multiple runs, to ensure that most of the local minima are found that increases the chance of finding the global minima. Between these two options, increasing **N** is better as the Tabu list ensures that the trial geometries are dissimilar, while multiple runs may end up in exploring the same local minima more often.

### 4.2. Binary Clusters

The mixing of two elements may result in properties that are different from the pure forms of each elemental clusters. In the case of binary clusters, we have to consider all different compositions between two elements. Here we have exhaustively explored all combinations in A_*i*_B_*j*_, where 1 ≤ *i* ≤ *m*; 1 ≤ *j* ≤ *n*; the cluster size *N* = *m* + *n*, for ruthenium-platinum and gold-platinum binary clusters. One notable feature in the geometries is that, one of the elements tends to become part of the core, while the other tends to be on the surface. The other property of interest is the mixing energy that shows the stability of binary clusters compared to that of the pure unary clusters.

#### 4.2.1. Ruthenium-Platinum Binary Clusters

**Figure 8:**
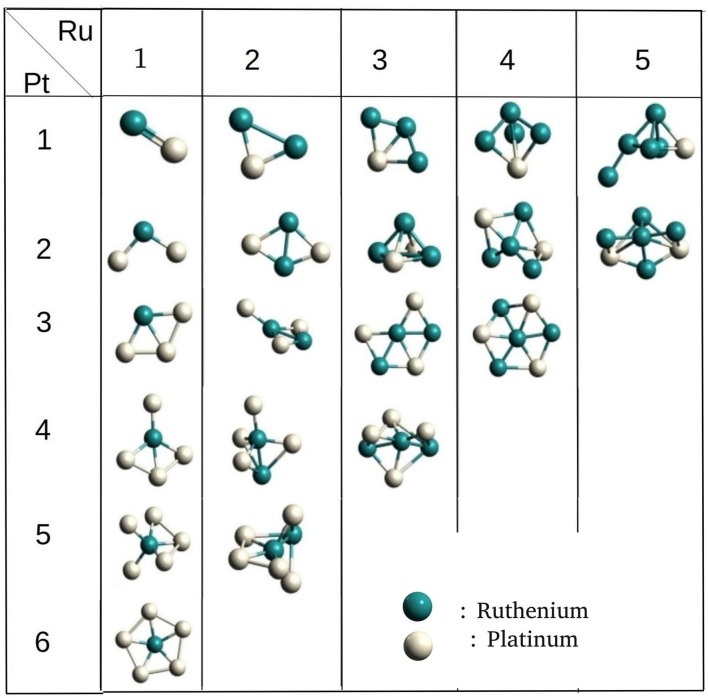
The optimized global minimum geometries of Ru-Pt binary clusters of size 2–7.

The binary Ru-Pt nanoalloys showed remarkable enhancement in catalytic activity for CO oxidation (Arico et al., 2001; Liu et al., 2006), compared to when platinum is used in its pure form as a catalyst (Bion et al., 2008), and avoids some of the drawbacks. We applied our method for building binary clusters implemented in binary_aggregator in PyAR to build Ru-Pt binary system with the interface to XTB program using GFN-xTB method. We built the Ru-Pt binary clusters up to a total cluster size of 14, i.e., Ru_1_Pt_1_ ⋯ Ru_7_Pt_7_. The lowest energetic clusters are shown in Figure 8 for a size of 2 to 7.

The general features of the GM geometries match with the reported trend (Demiroglu et al., 2017). In general, the Ru prefers to occupy the core of the clusters with the maximum number of bonds. The Pt, on the other hand, minimizes its number of bonds by seeping on to the surface, having at most three bonds. This observation is in accordance with the higher cohesive energy of Ru (6.74 eV) compared to Pt. (5.84 eV) (Kittel, 2005). The binding energy of the Ru_2_ dimer is lower than that of Pt_2_, 2.00 eV and 1.94 eV, and the Ru-Pt has the higher binding energy than both (2.13 eV) (Demiroglu et al., 2017). The GM geometries from PyAR|XTB maintain these qualitative features although the individual structures are not identical with the reported structures from Demiroglu et al., as most of these geometries have high spin ground states and we have considered only singlet states (Demiroglu et al., 2017).

For the cluster size of four, all the combinations of (Ru,Pt)_4_ have similar, non-planar bitriangular geometries. Ru-Ru bond is shorter in Ru_2_Pt_2_, but two Pt atoms prefer to stay away from each other. For cluster size higher than four, the geometry of GM changes with composition. As the composition of Ru increases in (Ru, Pt)_5_, the structure changes from planar to 3-D. Similar planar structures were found for Ru_1_Pt_4_ and Ru_2_Pt_3_. For cluster sizes with six and seven atoms also, the clusters with a higher composition of Ru have 3-D structures.

**Figure 9:**
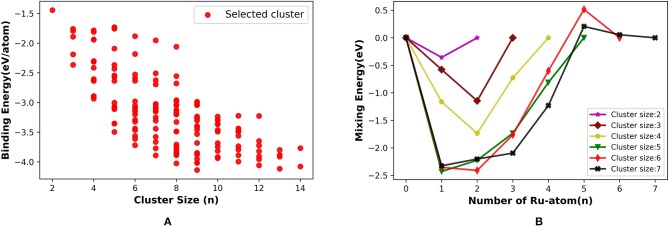
**(A)** Binding energy per atom (eV/atom) with increasing size from 2 to 14 and **(B)** mixing Energy vs. number of Ru atom for Ru-Pt binary clusters.

The binding energy per atom increases with the cluster size for (Ru, Pt)_N_ binary cluster in the range that we have considered, up to total cluster size 14. Figure 9A shows the average binding energy vs. cluster size of the Ru-Pt clusters, which includes all the selected unique isomers along with GM. The highest BE for each cluster size increases as the cluster grows and gains the highest stability at nine and then again at 13. Our semi-empirical results are in qualitative agreement with the reported DFT results (Demiroglu et al., 2017).

We have calculated the mixing energy (δ), the excess energy of nanoalloy over the pure cluster of the same size, for RuPt binary clusters of size **N** = m + n = 2–7. The effect of mixing Ru with Pt in small clusters calculated as a function of Ru atoms for all compositions of Ru_m_Pt_n_ from 2 ⩽ *N* ⩽ 7 clusters are plotted in Figure 9B. The mixing is favorable when δ is negative. In our calculation, mixed clusters are more stabilized than the pure clusters except for Ru_5_Pt_1_ and Ru_5_Pt_2_. The DFT calculation by Demiroglu et al. (2017), on the other hand, shown positive mixing energy for Ru_3_Pt_1_. Ru-Pt diatomic molecule is more stable than the pure Ru_2_ or Pt_2_ dimer. The clusters with one Ru atom (Ru_1_Pt_N_) more stable than the other possible combinations for ***N*** = 2, 5, and 7. Two Ru atoms made the binary clusters more feasible when ***N*** = 3, 4, and 6. Therefore, (Ru, Pt)_N_ binary clusters with a lesser composition of Ru atoms (one or two) are more favorable in our calculation using semi-empirical method (PyAR|GFN-xTB).

#### 4.2.2. Platinum-Gold Binary Clusters

Platinum-Gold nanoalloys are one of the most studied binary clusters because of their catalytic properties, for example, as a catalyst for CO adsorption (Logsdail et al., 2009; Kaizuka et al., 2010). Song et al. have studied the bonding properties of CO on Pt-Au binary clusters (Song et al., 2005). The catalytic activity of a cluster largely depends on the electronic properties. By introducing gold atom in the pure platinum cluster, the electronic properties and thereby, catalytic activity is enhanced.

**Figure 10:**
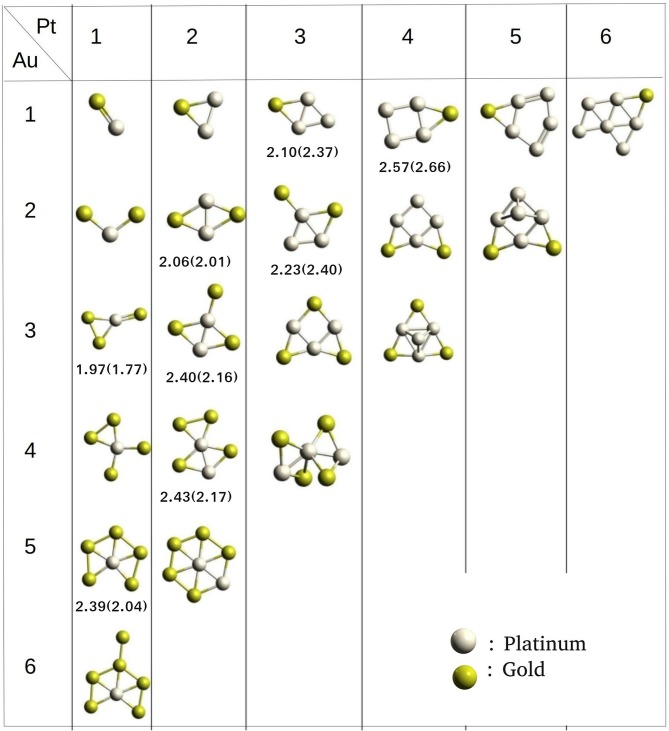
Optimized geometries of Pt-Au clusters from size 2–7 obtained using PyAR|XTB program. Binding energies (eV/atom) from GFN-xTB, and from DFT (PW91/PAW) result (Song et al., 2005) in parathesis.

We have built the (Pt, Au)_N_ binary clusters; *N* = 2–14 using PyAR|XTB. The lowest energy structures of *N* = 2–7 are shown in Figure 10. For (Pt,Au)_3_ cluster, the Pt_2_Au has a triangular geometry with Pt-Pt and Pt-Au bonds, while the PtAu_2_ has a bent structure with both the Au atoms bonded to Pt and has long Au-Au distance. PtAu_3_ has a planar structure with a triangle of PtAu_2_ and an exocyclic Au attached to Pt. The other (Pt,Au)_4_ structures, Pt_2_Au_2_ and Pt_3_Au_1_ has similar bicyclic quasi-planar structures. Among the (Pt,Au)_5_ clusters, Au_2_Pt_3_and Au_3_Pt_2_ where the composition of either gold or platinum is 60% have similar geometries as GM. The (Pt,Au)_5_ with 80% gold composition makes the structure having a triangular base, but the higher percentage of platinum changes the geometry to a fused four-and-three-membered rings.

As the cluster grows in size, the composition of the alloy will show significant effects on the structure and other properties. For cluster sizes of six and seven atoms, the structures with a higher composition of gold prefer to form planar-like structure. When the composition of Pt is maximum, the cluster tends to acquire a 3D geometry. While Au occupies external sites, Pt occupies the core sites. Apart from these general features, the GM geometries from our study do not match well with the global minimum geometries reported in the literature (Song et al., 2005), due to the different level of theory applied (GFN-xTB vs PW91/PAW) for studying the clusters.

**Figure 11:**
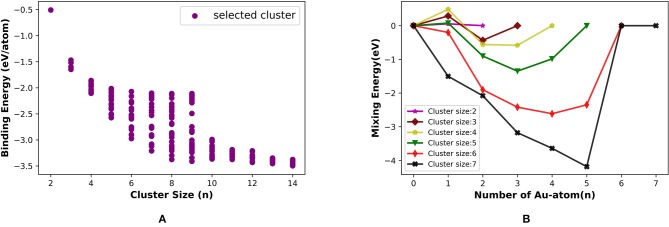
**(A)** Binding energy per atom (eV/atom) with increasing size from 2 to 14 and **(B)** mixing energy vs. number of Au atom for Pt-Au binary clusters.

We estimated the average binding energy for (Pt, Au)_N_ clusters (*N* = 2–14) using PyAR|GFN-xTB. The cluster gains the highest stability when it reaches the size nine and again at size 14 in Figure 11A. We compared our results (PyAR|XTB) with the DFT results by Song et al. (2005). Binding energies of planar Pt_1_Au_3_, Pt_2_Au_2_, and Pt_3_Au_1_ are shown in 10 with the corresponding reported values. The planar minima of Pt_4_Au_1_, Pt_3_Au_2_, and Pt_2_Au_3_ are in agreement with the reported geometries.

We have calculated the mixing energy—the stability of mixed cluster compared to its pure form—for the (Pt, Au)_N_ clusters. Most of the GM geometries with combinations of Pt_m_Au_n_ (2 ⩽ *m* + *n* ⩽ 7) clusters have negative mixing energy, except for Pt_1_Au_2_, Pt_1_Au_3_ (Figure 11B). Hence, the mixing is, in general, favorable. For cluster size up to seven, the clusters with one or two Pt atoms have the highest stability.

### 4.3. Ternary Aggregate

The catalytic activity of metal nanoclusters can be enhanced by introducing a second element as well as a third element. Some ternary metal clusters were shown to have higher activity than their unary or binary counterparts (Fang et al., 2011). However, the details of the mechanisms for the enhanced activity is largely unknown as even the structural details of these binary, ternary or other heterometallic clusters are unknown. Finding the global minima of the ternary cluster is even more difficult as compared to the unary and binary systems. Ternary cluster, A_l_B_m_C_n_ (l + m + n = N), can have geometries different from their unary or binary counterparts and can have different structures for different compositions. This high level of complexity in the PES is a challenge for the high-level theoretical calculations to explore the surfaces efficiently. There is a lot to be learnt about the ternary clusters; computational chemistry can serve greatly in this endeavor.

**Figure 12:**
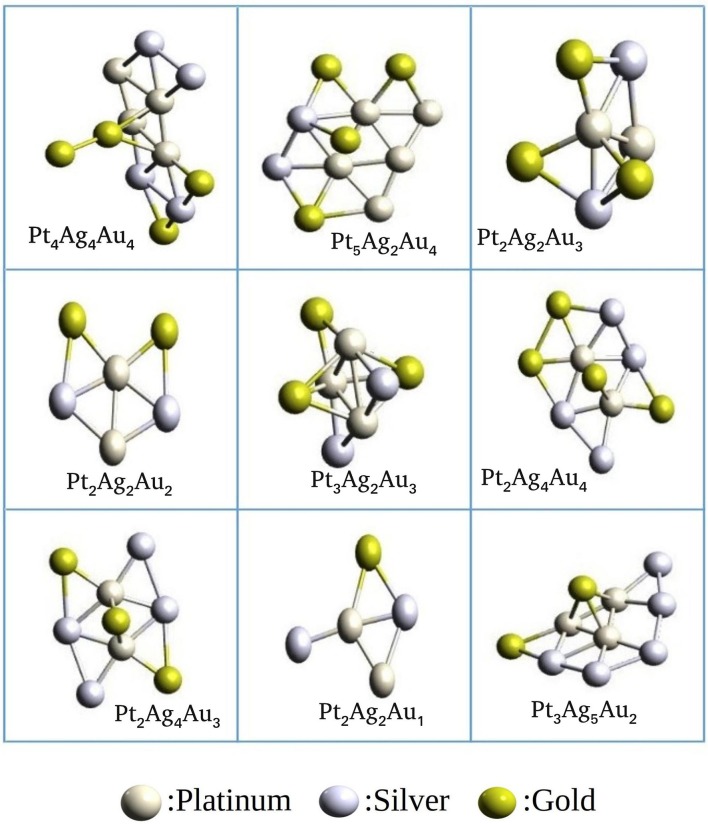
Ternary clusters of Ag, Au, and Pt generated using PyAR|XTB program.

We have studied Platinum-Gold-Silver clusters as an example for a ternary system to validate the ternary_aggregator module implemented in PyAR program. We have built the Pt-Ag-Au cluster of total size up to 15 using xTB interface. In the GM geometries, Pt and Ag are near the core, while Au atoms are in the periphery. As we have discussed in the binary systems, these preferences can be attributed to the bond strengths. The bond strength calculated at GFN-xTB level follows the order: Ag-Ag (-5.19 eV) > Pt-Pt (-4.4 eV)> Au-Au(-3.9 eV); the bond energy is given in parenthesis. The geometries of the most stable ternary clusters are shown in Figure 12. Most of the structures are quasi-planar or three-dimensional. The general features of the minima are in accordance with the studies by Pacheco-Contreras et al. using Basin Hopping global search with Gupta Potential (as the force field), and using DFT for final optimization for more accuracy (Pacheco-Contreras et al., 2018).

## 5. Conclusions

We have developed a methodology to build the unique geometries of nanoclusters and nanoalloys. The clusters are built by adding atoms one-by-one starting from one atom up to the desired size. The following steps are involved in the method: (a) For adding an atom to the N-sized cluster, several trial orientations are generated by placing the atom at different random positions around the cluster, (b) These orientations are then optimized by gradient-based methods by the interfaced electronic structure programs, (c) From all the optimized geometries, the similar geometries are removed, the unique structures are selected by clustering algorithms, and these selected geometries are used for the next cycle. These steps (a–c) are repeated to add an atom to all the selected seed molecules. This atoms are added until the cluster grows to the desired size. The similarity between the molecules are compared using the molecular representation based on fingerprints of the Coulomb matrix.

We studied nanoclusters of palladium, gold, platinum, and aluminum, binary clusters of Ru-Pt and Au-Pt, and ternary clusters of Ag-Au-Pt. The method is shown to produce all the reported global minimum structures, along with other minima, when we used the same or similar electronic structure methods and the same spin-states. Differences were seen when we used the semiempirical GFN-xTB method to compare the reported structure and properties from DFT or CCSD(T) studies. We have also evaluated some popular properties such as binding energy per atom, mixing energy, and compared with the reported ones.

We have varied some of the parameters in our approach for comparison of efficiency in finding the global minima and other properties of metal nanoclusters. We have compared different electronic structure methods, semiempirical and a few DFT functionals, in gold and aluminum clusters. While all the methods produced the same global minima for gold clusters, the geometries of maximum stability were highly dependent on the method for Al clusters. The method dependency was also seen in identifying the ground spin-state in Pt clusters. Thus, we can use less expensive methods such as semiempirical methods or empirical potentials for the clusters which do not change the ground-state multiplicity, and for which these methods give good results comparable to high-level *ab initio* or DFT methods. We can also use a two-layer approach where the initial search is done by cheaper methods, and the selected geometries can be optimized at a higher level.

We checked the effect of varying the number of orientations by comparing the binding energy per atom in the Au clusters. The study showed that as the cluster size increase more orientations has to be used for better results. In the same way, the result from different runs may vary if a small number of orientations are used, as was found by comparing the BE for three separate runs for Pt and Al clusters. As the cluster size increases, the search space increases and hence either number of orientations has to be increased or multiple runs have to be carried out to ensure that most of the local minima are found to increase the chance of finding the global minima.

A major potential challenge in such cluster-growing methods is the ability to capture the changes in structural motifs. We have seen that the GM's in Au_n_ clusters changed from 2D to 3D on going from *n* = 10–11. We also found similar structural changes in carbon clusters, 1D linear geometry, to 2D ring structures, and 3D structures including bowl-shaped geometries.

Thus, by building the cluster of size *n* by exploring three degrees of freedom involving the relative orientations of *n*−1 and one atom gives the minima obtained by exploring 3N-6 degrees of freedom by the other methods. The direct comparison of complexity for the *n*-sized cluster is not meaningful because we have to add the complexity for exploring each of the clusters of size up to *n*. The major limitation our method is that it can be more expensive for building a cluster of a particular size of *n* as the method has to build all the clusters from size 2 to *n*. This method may not be very useful if one is only interested in only the *n*-sized cluster. Our method, however, is useful for studying the evolution of properties with growing cluster size.

## Data Availability Statement

All datasets generated for this study are included in the manuscript/Supplementary Files.

## Author Contributions

AA designed the project. MK and RM did the calculations. All the authors contributed to the manuscript preparation.

### Conflict of Interest

The authors declare that the research was conducted in the absence of any commercial or financial relationships that could be construed as a potential conflict of interest.
