# Supplementary material for: A Global Optimizer for Nanoclusters
Source: Front Chem. 2019 Sep 27;7:644. doi: 10.3389/fchem.2019.00644 (PMC6776882; doi:10.3389/fchem.2019.00644)
Supplement: Supplementary file 1 [file Data_Sheet_1.PDF]

A Global Optimizer for nanoclusters

SUPPORTING INFORMATION

Homometallic Clusters

1. Palladium Clusters

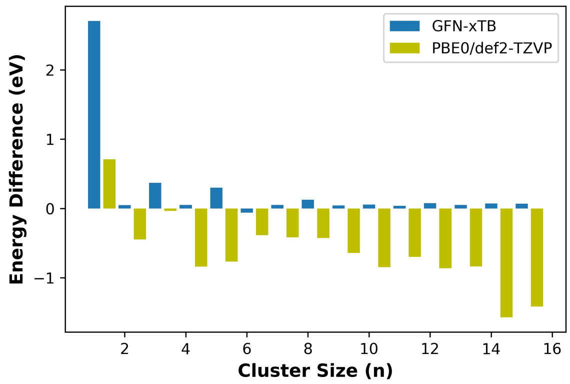

Figure S1. The singlet triplet energy difference (ΔE_{ST} ; in eV) of Pd_n clusters obtained using PyAR|XTB and the geometries further optimized at PBE0/def2-TZVP methods

2. Gold Clusters

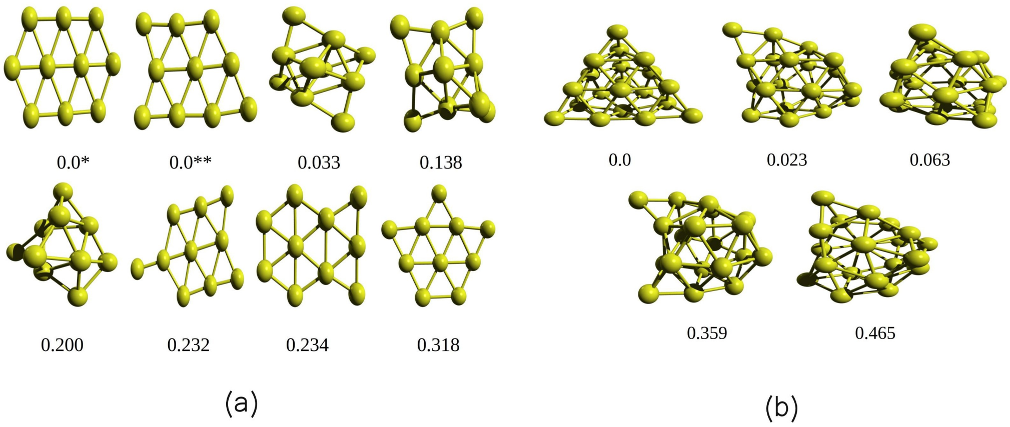

Figure S2. a) Structures of Au_{10} below 0.4 eV b)Structures of Au_{20} below 0.5 eV obtained by the global search using PyAR.

3. Carbon Clusters

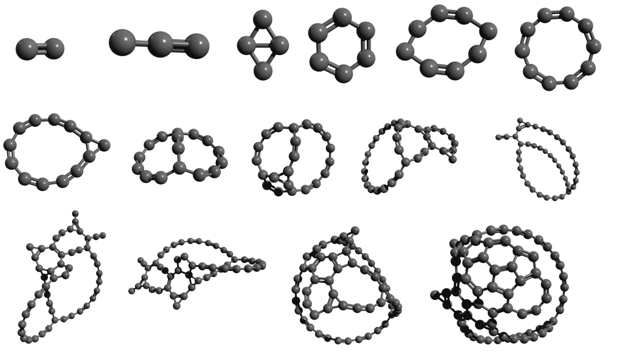

Figure S3. The global minimum geometreis of selected carbon clusters

CARTESIAN COORDINATES

OPTIMIZED GEOMETRIES

```
Pd_1
1
Pd      0.000000      0.000000      0.000000

Pd_2
2
SCF Energy =  -255.735167
Pd      0.000000      0.000000     -0.381859
Pd      0.000000      0.000000     -3.118141

Pd_3
3
SCF Energy =  -383.662641
Pd      0.619772      0.000000      1.979063
Pd     -1.138914      0.000000      0.269273
Pd     -1.740158      0.000000      2.656314

Pd_4
4
SCF Energy =  -511.596657
Pd      1.319643     -0.729485     -0.346427
Pd      0.271728      1.188059     -1.596897
Pd     -1.398301     -0.476664     -0.749014
Pd      0.164441     -1.414300     -2.501942

Pd_5
5
SCF Energy =  -639.549046
Pd      0.360389      1.020675      1.459059
Pd      1.973000     -0.216988     -0.270077
Pd     -0.537060     -0.616702     -0.672056
Pd     -1.543205     -0.603958      1.771932
Pd      0.796466     -1.793837      1.367741

Pd_6
6
SCF Energy =  -767.464069
Pd      0.278657     -0.526557      0.879533
Pd      0.294126      2.109680      0.886038
Pd     -1.811669      0.809830     -0.003154
Pd     -0.303121     -0.513357     -1.717681
Pd      1.802675      0.786508     -0.828498
Pd     -0.287656      2.122886     -1.711176

Pd_7
7
SCF Energy =  -895.396322
Pd     -0.890410      1.533769      0.333887
Pd      1.077191     -0.292800     -1.383706
Pd     -1.233187      0.552426     -1.994911
Pd     -1.047047     -1.194859      0.069605
Pd      1.779975      1.939020     -0.229581
Pd      1.023402     -0.130105      1.336023
Pd      0.094299      2.771420     -1.976107

Pd_8
8
SCF Energy = -1023.308433
Pd      0.605408      1.897125      0.813723
Pd      1.874730     -0.720812      0.452134
Pd     -1.393606      0.462500     -0.001724
Pd     -0.178049     -1.758150     -0.831650
Pd      0.778884      0.670323     -1.541573
Pd     -0.187508     -1.381573      1.846069
Pd     -1.096497      1.022565      2.607217
Pd      1.513847      0.401891      2.838155

Pd_9
9
SCF Energy = -1151.241932
Pd     -0.507007      0.856307      0.723956
Pd      1.126051     -1.369670     -0.319349
Pd     -0.343045      0.166680     -2.041116
Pd     -1.580357     -1.268464     -0.139805
Pd      1.956536      1.101393     -0.875981
Pd     -0.101354     -1.208792      2.109063
Pd     -0.256552      2.651506     -1.037029
Pd      2.112752      0.212397      1.688522
Pd      1.172404      2.672534      1.234067

Pd_10
10
SCF Energy = -1279.165809
Pd      0.898603      0.560459      1.292268
Pd      1.441407     -1.495814     -0.480807
Pd     -1.247401     -0.164901     -0.693360
Pd     -1.001221     -2.663671     -0.989174
Pd      1.215949      1.099694     -1.376686
Pd     -0.304538     -1.891434      1.597478
Pd     -1.808470      0.435667      1.712166
Pd      0.046770     -0.870374     -2.743698
Pd     -0.796693      2.232671     -0.068877
Pd     -2.872797     -1.900258      0.779890

Pd_11
11
SCF Energy = -1407.106237
Pd     -0.249875      1.035262      0.506013
Pd      0.982018     -1.471092      0.007354
Pd     -1.274921     -0.353488     -1.440202
Pd     -0.937387     -3.000542     -0.716906
Pd      1.304442      0.622224     -1.726924
Pd     -1.581392     -1.067881      1.102129
Pd      0.877183     -0.691137      2.414762
Pd      0.721815     -1.916844     -2.448717
Pd      1.471548      2.768945     -0.103160
Pd      1.174822      2.030154      2.390392
Pd      2.616745      0.429559      0.716219

Pd_12
```

```
12
SCF Energy = -1535.034688
Pd      1.438776      1.598439     -0.268773
Pd      1.599891     -0.948011     -1.328561
Pd     -1.004749      0.475693     -0.655103
Pd     -0.519255     -2.199527      0.063391
Pd      0.370791      1.032274     -2.717809
Pd      0.945890     -0.393078      1.228822
Pd     -0.649870      1.769990      1.681345
Pd     -1.976050     -0.401710      1.522522
Pd      1.113563      0.670538      3.515761
Pd     -1.519390      0.564064      3.946444
Pd     -0.843872     -1.290871     -2.493391
Pd     -0.140066     -1.675081      3.071840

Pd_13
13
SCF Energy = -1662.972954
Pd      1.438689      1.235682     -0.170757
Pd      1.457706     -1.243452     -1.533215
Pd     -1.048009      0.580284     -1.321745
Pd     -1.087510     -2.155659     -0.817956
Pd      0.956395      1.062581     -2.799538
Pd      0.243621     -0.707615      0.835153
Pd     -1.025973      1.866931      0.894650
Pd     -2.575009     -0.284160      0.578220
Pd      1.079115      1.206670      2.495593
Pd     -1.409927      0.171391      3.017919
Pd     -0.627001     -1.051060     -3.230845
Pd      0.807571     -1.369646      3.345449
Pd     -1.293165     -2.335746      1.948792

Pd_14
14
SCF Energy = -1790.903025
Pd      1.252326      1.188025      2.085091
Pd      2.504490     -0.806893      0.795875
Pd      0.010516     -0.013048      0.085779
Pd      0.567787     -2.618891     -0.261004
Pd      1.999082      1.757679     -0.331977
Pd      0.338352     -1.664372      2.143401
Pd     -1.680968      0.582354      2.037782
Pd      1.556737     -0.530386     -1.998451
Pd     -0.562642      2.584990      0.633531
Pd     -1.957422     -1.740234      0.769245
Pd     -1.106821     -1.115585     -2.021859
Pd     -2.513053      0.809703     -0.402958
Pd     -0.271028      1.482284     -2.083147
Pd     -0.091470      0.077246      3.948482

Pd_15
15
SCF Energy = -1918.845009
Pd      1.810645      2.130056     -0.280589
Pd      2.305974     -1.037624     -1.947107
Pd     -1.438777      0.858993     -1.895382
Pd     -2.205725     -1.756213     -1.492801
```

```
Pd      1.182973      1.149808     -2.577591
Pd      0.105370     -0.238548     -0.264598
Pd     -0.718402      1.944547      0.683874
Pd     -2.664551     -0.040415      0.459690
Pd      1.418943      1.127679      2.213786
Pd     -1.032742     -0.071084      2.492320
Pd     -0.110821     -1.150637     -3.216806
Pd      1.091196     -1.557463      1.609558
Pd     -1.565937     -2.186248      1.023184
Pd      2.963860     -0.048887      0.342514
Pd      0.310970     -2.637302     -0.984744

Au_3
3
SCF Energy =-11.8583272811
Au      0.88837      1.01555      0.26370
Au     -0.35453     -1.33062      0.12510
Au      1.95888     -1.12623      1.41924

Au_4
4
SCF Energy =-15.8573539487
Au     -0.24506      1.55027      0.33447
Au     -1.41382     -0.78998      0.19477
Au      1.29397     -0.67332      0.14776
Au     -2.95285      1.43362      0.38144

Au_5
5
SCF Energy =-19.8418394657
Au      0.44796      0.40853      1.31288
Au     -0.51916     -0.81982     -0.97650
Au      2.08774     -0.84644     -0.26965
Au     -2.24864      0.49316      0.67519
Au     -1.21062      1.65762      2.88038

Au_6
6
SCF Energy =-23.8512113801
Au      0.75831      0.68137      0.30790
Au     -0.33026     -1.79444     -0.50012
Au      2.03754     -0.96719     -1.29450
Au     -1.79357     -0.10442      1.22326
Au     -0.68782      2.18039      1.91522
Au     -2.71524     -2.43069      0.41033

Au_7
7
SCF Energy =-27.8210482223
Au      1.02896      1.05616     -0.07287
Au      0.11982     -1.51902     -0.76295
Au      2.16471     -0.33190     -1.97393
Au     -1.08843      0.19901      1.40420
Au      0.00450      2.56239      1.79439
Au     -1.94091     -2.25479      0.73367
Au     -3.13488     -0.84694      2.70119
```

```
Au_8
8
SCF Energy =-31.8288355606
Au      1.12175      0.92030     -0.54095
Au      1.19137     -1.82986     -0.52404
Au      2.90025     -0.41845     -1.91194
Au     -1.04885      0.87525      1.14907
Au     -0.00953      3.12137      0.30414
Au     -0.97866     -1.87536      1.16556
Au     -2.77884     -0.53702      2.50985
Au      0.17324     -4.07539      0.34794

Au_9
9
SCF Energy =-35.8008191447
Au      1.18973      1.62670     -1.21545
Au      0.55119     -1.02284     -0.95868
Au      2.43201     -0.19454     -2.73321
Au     -0.78684      1.00618      0.68570
Au      0.02291      3.48423      0.23147
Au     -1.36129     -1.74059      0.86367
Au     -2.59028      0.03494      2.36107
Au     -0.16307     -3.62012     -0.61807
Au      1.78326     -2.89123     -2.47322

Au_10
10
SCF Energy =-39.8054548362
Au      1.03920      2.13288     -1.03865
Au      0.36709     -0.51323     -0.52200
Au      2.42551      0.12513     -2.19251
Au     -1.06235      1.50516      0.66723
Au     -0.38593      4.09482      0.14631
Au     -1.73447     -1.14095      1.18388
Au     -3.12068      0.86680      2.33784
Au     -0.30942     -3.10288     -0.00119
Au      1.77456     -2.50062     -1.69296
Au     -2.46983      3.49253      1.83819

Au_11
11
SCF Energy =-43.7875247098
Au      1.50332      1.17413     -0.26209
Au      2.41593     -1.43588      0.15827
Au      1.82062     -0.53355     -2.55302
Au     -1.22296      1.46546      0.93851
Au     -0.53490      2.84860     -1.20758
Au     -1.54245     -1.43130      0.58116
Au     -1.78633     -0.08655      3.00590
Au      0.07786     -2.49461     -1.27367
Au      0.79921     -0.37094      2.01530
Au     -0.84971      0.10864     -1.70320
Au      2.65317     -3.10391     -2.01197

Au_12
12
SCF Energy =-47.7960503542
Au      0.79512      1.19394      0.83782
Au      2.22326     -1.11999      0.19346
Au      1.15356      0.33277     -2.08989
Au     -1.73402      2.57844      0.89597
Au     -0.18872      2.53733     -1.51683
Au     -1.56812     -2.12093      0.97915
Au     -1.72711      0.40935      2.40769
Au     -0.11641     -2.15958     -1.28739
Au      0.77155     -1.08138      2.46003
Au     -1.55437      0.15009     -0.64919
Au      2.40194     -2.01281     -2.41569
Au     -1.53707     -1.90802      3.73422

Au_13
13
SCF Energy =-51.7806678132
Au      1.25165      1.45271      0.62440
Au      2.47265     -0.97196     -0.17000
Au      1.17159      0.64107     -2.22916
Au     -1.27387      2.81959      1.03891
Au     -0.01430      2.90672     -1.51848
Au     -1.28916     -1.79775      0.95037
Au     -2.59398      0.53161      1.91682
Au     -0.05119     -1.89337     -1.44384
Au      1.22787     -0.87830      2.22073
Au     -1.36604      0.49671     -0.69665
Au      2.32607     -1.67789     -2.83925
Au     -1.15375     -1.15383      3.67730
Au     -0.12588      1.43333      3.16232

Au_14
14
SCF Energy =-55.7869258694
Au      1.93497      1.14494      0.84774
Au      2.28319     -1.31274     -0.40671
Au      0.89140      1.03929     -1.92422
Au     -0.45606      2.41239      0.21126
Au     -1.25396      2.72050     -2.40899
Au     -1.45797     -1.73016      0.58911
Au     -2.63873      0.80452      1.61635
Au     -0.22293     -1.65828     -1.82471
Au      1.03381     -1.36741      1.97153
Au     -2.09450      0.23496     -1.24307
Au      2.15150     -1.06623     -3.11917
Au     -1.40022     -1.12269      3.29759
Au     -0.10991      1.32516      2.70773
Au     -3.24452      2.59987     -0.37360

Au_15
15
SCF Energy =-59.7812970245
Au      1.50977      1.09911      0.33517
Au      2.58914     -1.43975     -0.30013
Au      1.45836      0.25818     -2.45856
Au     -0.05460      3.36837      0.98218
Au      0.21106      2.52664     -1.85357
Au     -1.17105     -2.13496      0.58827
```

```
Au    -2.52629     0.32990     1.24563
Au     0.26670    -2.34860    -1.68059
Au     1.15897    -1.22325     1.97311
Au    -1.20007     0.03883    -1.27544
Au     2.68105    -2.11074    -2.95615
Au    -1.34066    -1.24244     3.23862
Au    -0.19627     1.24162     2.63055
Au    -2.37701     2.45957    -0.39829
Au    -1.10746     4.77958    -1.11848

Au_16
16
SCF Energy =-63.786172337
Au     2.16519     0.73001     0.32314
Au     2.23466    -2.07570    -0.08935
Au     1.50458    -0.16686    -2.28434
Au     0.44461     2.98628     1.28710
Au     0.39346     2.10800    -1.49282
Au    -0.71434    -2.51397     0.64163
Au    -2.66468    -0.41204     1.22074
Au     0.02229    -2.67018    -1.97304
Au     1.22152    -1.11654     2.22563
Au    -1.30015    -0.34433    -1.24839
Au     2.67964    -2.57116    -2.73480
Au    -1.29587    -1.78634     3.28015
Au    -0.82190     0.95282     2.80009
Au    -2.06401     1.99055     0.19873
Au    -0.90002     4.34578    -0.57444
Au     1.89067     1.58052     3.04168

Au_17
17
SCF Energy =-67.7860820965
Au     1.94380     0.64521     0.02719
Au     1.89499    -2.17655    -0.37828
Au     1.20242    -0.23234    -2.57875
Au     0.35051     2.88517     0.96220
Au     0.18993     2.09487    -1.79791
Au    -0.80191    -2.87459     0.52591
Au    -2.72128    -0.63274     0.64397
Au    -0.33716    -2.64427    -2.29388
Au     0.94204    -1.23436     1.98260
Au    -1.64828    -0.07253    -1.98185
Au     2.23256    -2.71931    -3.05579
Au    -1.64354    -1.82008     2.97800
Au    -1.10303     0.85968     2.37825
Au    -2.28054     1.94836    -0.07259
Au    -1.07188     4.30928    -0.80922
Au     1.64939     1.36970     2.77239
Au    -2.87049    -2.58047    -1.37823

Au_18
18
SCF Energy =-71.7996117842
Au     2.03498     0.73442     0.33314
Au     2.16361    -2.05404    -0.35596
Au     1.24852    -0.13594    -2.32126
Au     0.45596     2.95177     1.25878
Au     0.43328     2.25390    -1.55035
Au    -0.31160    -2.49740     1.14882
Au    -3.53309     0.37044     0.46247
Au    -0.38470    -2.46385    -1.78050
Au     2.04692    -1.45868     2.25519
Au    -1.56687     0.15520    -1.68035
Au     2.03186    -2.67171    -2.99312
Au    -0.34798    -1.16680     3.70697
Au    -1.52370     1.05580     2.29499
Au    -2.09235     2.54200    -0.16579
Au    -0.47150     4.66674    -0.62596
Au     1.16522     1.12594     3.08892
Au    -2.65137    -2.21423    -0.37124
Au    -2.75035    -1.55312     2.36403

Au_19
19
SCF Energy =-75.7978483877
Au     2.23425     0.46427     0.03203
Au     2.29578    -2.26497    -0.90526
Au     1.42172    -0.10284    -2.67889
Au     0.84394     2.68985     1.23409
Au     0.73101     2.25439    -1.66110
Au    -0.10016    -2.72455     0.60272
Au    -3.25694     0.34856     0.26365
Au    -0.27000    -2.44698    -2.28271
Au     2.39826    -1.86981     1.72211
Au    -1.29480     0.23044    -1.82136
Au     2.10986    -2.56742    -3.60233
Au    -0.05286    -1.60978     3.28351
Au    -1.14041     0.82558     2.13556
Au    -1.73334     2.54702    -0.12644
Au     0.00739     4.60778    -0.50430
Au     1.63713     0.72774     2.84367
Au    -2.52304    -2.15607    -0.81777
Au    -2.41414    -1.73620     1.97382
Au     2.42179    -1.36457     4.39500

Au_20
20
SCF Energy =-79.8114596528
Au     1.96916     0.71302    -0.14936
Au     2.34038    -1.96472    -1.13848
Au     1.34725     0.15213    -2.90347
Au     0.39272     2.80856     1.03811
Au     0.41920     2.42653    -1.86635
Au    -0.08129    -2.62462     0.27097
Au    -3.51293     0.18450    -0.12986
Au    -0.17748    -2.32982    -2.59094
Au     2.31626    -1.61702     1.50501
Au    -1.41687     0.22207    -2.10255
Au     2.23495    -2.24029    -3.84102
Au    -0.22811    -1.60006     2.95707
Au    -1.45245     0.73580     1.80331
Au    -2.12517     2.44349    -0.41433
Au    -0.58251     4.66463    -0.69241
```

```
Au     1.29662     0.88190     2.64451
Au    -2.49330    -2.31438    -1.26933
Au    -2.51980    -1.93235     1.63512
Au     2.16174    -1.18465     4.18458
Au    -4.79565    -2.19345     0.17123

Au_10
10
SCF Energy =-39.73399337
Au    -2.04415    -0.86026    -2.30355
Au    -0.00612    -2.21053    -1.16002
Au    -0.61687     0.37091    -0.33316
Au    -2.66353     1.69617    -1.49806
Au     1.46169    -0.97505     0.84987
Au     2.88885     0.25616     2.82022
Au     0.85094     1.60638     1.67673
Au    -1.19906     2.90324     0.49348
Au     2.04386    -3.50742     0.02315
Au     3.50827    -2.30031     2.01471

Au_10
10
SCF Energy =-39.7332151
Au     0.97274    -0.64823    -2.83755
Au     0.54905    -0.19196     0.07477
Au    -1.85166     0.76190     0.84718
Au     0.29190     0.16899     2.79148
Au     2.90378    -1.15752    -0.84127
Au    -1.42237     0.34766    -1.73210
Au     2.61767    -0.77792     1.80961
Au    -1.08608    -0.03580    -4.32956
Au    -2.00833     1.08357     3.53617
Au     3.40314    -1.60490    -3.36532

Au_10
10
SCF Energy =-39.7319629
Au    -1.25649    -2.50473     1.17426
Au     2.48037     1.34865     0.68575
Au    -0.15814     2.06740     0.21349
Au     1.26963     0.44742    -1.64316
Au    -0.70304    -1.58626    -1.38543
Au    -2.13064     0.03362     0.47088
Au     1.48501    -1.91178    -2.90764
Au     0.70802    -0.65356     1.06981
Au    -2.22271     2.29548     1.91542
Au    -1.49217     1.07467    -2.13026

Au_10
10
SCF Energy =-39.728325
Au    -2.28236    -2.08647     1.92847
Au     0.25364    -1.32561     1.84691
Au    -1.74855    -0.00044     0.42617
Au     2.20461    -0.27338     0.12920
Au     0.23772     1.07375    -1.32028
Au     2.82828    -1.72910     2.23499
Au     2.58748     0.53995    -2.36279
Au    -2.43152     1.31270    -1.87479
Au    -1.34508     2.75896     0.24792
Au     0.58292     1.57217     1.70700

Au_10
10
SCF Energy =-39.7257731
Au    -1.38366    -1.88646     1.48119
Au     2.77621     1.16607     1.13315
Au     0.12512     1.55964     0.80705
Au     1.74690     0.28334    -1.23272
Au    -0.38326    -1.70417    -1.00999
Au    -2.22872     0.40324    -0.30283
Au     0.35174    -0.51844    -3.33524
Au     1.18044    -1.08059     1.24670
Au    -2.26598     2.50891     1.30658
Au    -2.45990    -0.83054    -2.64202

Au_10
10
SCF Energy =-39.7246092
Au     0.33575    -1.21271    -1.74125
Au     0.39040     1.31216     2.14226
Au    -0.93389     0.55319    -0.10872
Au     1.81901    -0.51566     0.54759
Au     2.70695    -2.20606    -1.28635
Au     2.76475     0.46269     2.81854
Au    -2.07242    -0.31957    -2.37978
Au    -2.01593     2.28892     1.63233
Au    -3.37479     1.50208    -0.69163
Au    -5.66343     2.39449    -1.23494

Au_10
10
SCF Energy =-39.72453
Au    -1.30566    -0.78684    -0.05233
Au    -0.19694     1.63157     0.63741
Au    -2.64215     0.90140     1.55441
Au     0.22320    -2.39235    -1.71640
Au    -2.10992    -3.17620    -0.88944
Au     1.32933     0.05013    -1.01283
Au     2.56505    -1.69204    -2.59222
Au     2.23873     2.45274    -0.22647
Au    -1.62749     3.32039     2.29454
Au     0.80066     4.09229     1.40833

Au_10
10
SCF Energy =-39.7214172
Au    -1.18569    -0.71686     0.41460
Au     0.25076     1.57275     1.35111
Au    -2.27040     0.87460     2.36337
Au    -0.06221    -2.28308    -1.53309
Au    -2.43559    -2.93310    -0.57100
Au     1.38347    -0.04754    -0.64936
Au     2.27536    -1.69953    -2.52641
```

```
Au     2.60859     2.08656     0.29326
Au    -0.98894     2.96805     3.23888
Au    -3.62480    -1.23278     1.52390

Au_20
20
SCF Energy =-79.7281054
Au    -0.78888    -1.60902     2.74984
Au     1.32728    -2.63861     1.00503
Au    -2.28623    -0.04085     0.85635
Au     3.05407    -0.95516    -0.13259
Au     2.16076     0.83520    -2.27251
Au     1.46633     0.03881     2.05318
Au     4.70126     0.86212    -1.30518
Au    -2.27628    -1.06831    -1.83269
Au    -1.88145     2.80186     1.06034
Au     0.90907     2.86115     1.95037
Au    -1.46322    -2.69782     0.11500
Au    -0.54104    -4.24862     2.14854
Au    -2.49525     1.81088    -1.33774
Au     2.83503     1.92397     0.36231
Au    -0.37907     0.78125    -3.08254
Au    -0.98814     1.01151     3.20024
Au     0.26504     2.44641    -0.82446
Au     0.55948    -1.42538    -1.49004
Au    -3.00944     0.69843    -3.76441
Au    -1.14612     3.70707     3.51609

Au_20
20
SCF Energy =-79.7272023
Au    -1.33879     2.74909     0.04591
Au    -0.94654     0.06208     2.56922
Au    -3.46948     1.06741     1.51430
Au     1.81258    -0.24708     2.76576
Au     2.84177     0.87857     0.32640
Au    -0.73450    -0.89414    -2.66264
Au    -2.78534     0.71286    -1.20929
Au     1.46186    -2.25080    -1.38285
Au    -1.63272     2.81525     2.85078
Au    -1.02854    -3.25150    -0.92461
Au     1.38055     2.89208    -0.83398
Au    -2.43435    -1.39971     0.72340
Au     0.24774    -2.14105     1.29288
Au     0.83565     2.23367     1.85556
Au     3.05982    -1.89370     0.93954
Au    -3.25014    -1.89849    -1.97235
Au    -0.64982     1.90871    -2.54311
Au     1.69405     0.46948    -2.34716
Au     3.89569    -1.00188    -1.60733
Au    -4.27100     2.84361     3.32601

Au_20
20
SCF Energy =-79.7257491
Au    -0.81393    -2.29389     2.08810
Au     2.18866     2.57113    -0.22190
Au    -0.62763     2.82806     0.48301
Au     0.01833     2.41544    -2.19405
Au     1.52519    -2.87705     0.80399
Au    -2.68291     1.27297     1.71853
Au    -0.28363    -0.39895    -2.72324
Au     2.58217    -0.20096     0.38882
Au     1.34994     1.59257     2.16870
Au    -2.13873     1.06742    -1.08689
Au    -0.66320     0.13844     3.56287
Au    -3.08052    -1.24624     3.19109
Au    -0.75616    -2.56337    -0.76927
Au    -2.99919    -1.19253     0.44769
Au     1.87573    -1.93670    -1.78457
Au     1.75687    -1.10625     2.94858
Au    -2.84887    -1.41144    -2.25085
Au     2.35042     0.65546    -2.39815
Au     2.58329     3.28282    -2.86853
Au    -0.91459     2.88292     3.25088

Au_20
20
SCF Energy =-79.7147781
Au    -0.82723    -2.18995     2.03918
Au     2.14135     2.43624    -0.45144
Au    -0.49919     2.31033     0.61286
Au    -0.24117     2.22152    -2.19772
Au     1.49368    -2.85282     0.72012
Au    -2.86264     1.24953     1.61188
Au    -0.47179    -0.59422    -3.10413
Au     2.73370    -0.34924     0.15236
Au     1.77663     1.59898     2.07906
Au    -2.30494     0.91006    -1.07875
Au    -0.54819     0.52747     3.07143
Au    -2.98451    -0.96430     3.25624
Au    -0.88157    -2.62046    -0.78494
Au    -3.13094    -1.39146     0.45875
Au     1.62619    -2.11804    -1.94313
Au     1.74037    -1.07129     2.82789
Au    -3.04963    -1.50708    -2.22313
Au     2.07774     0.43532    -2.55639
Au     2.14438     3.06591    -3.15708
Au    -2.53295    -1.06901    -4.78310

Au_20
20
SCF Energy =-79.7108587
Au     0.19281    -2.70473    -0.76067
Au     0.85328     0.10604     2.86363
Au     1.64249     1.81839    -1.36696
Au    -1.55580     1.05454     2.07391
Au     2.32342    -0.98763    -1.39692
Au    -0.53701     0.88703    -3.03192
Au     2.90163     0.74815     0.86889
Au    -3.20665    -0.61166     0.26444
Au    -2.44659    -3.28158    -0.01663
Au     0.46852    -1.73519    -3.45064
Au    -1.06219     2.76982    -1.08111
```

```
Au    -3.55073     2.12191     0.43868
Au    -0.94092    -1.65711     1.67606
Au    -1.65369     3.87528     1.40080
Au     1.96092    -2.01143     1.19713
Au     0.77908     2.53141     1.17132
Au     2.08635     0.57609    -3.79280
Au     3.43074    -0.74533     3.13441
Au    -2.00085    -1.53955    -2.18751
Au    -3.10083     1.04055    -2.11333

Pt_3
3
SCF Energy =-13.0332706
Pt    -0.56735    -1.14338    -0.40421
Pt     0.92715     0.32815     0.79920
Pt     0.97488    -2.07558     1.05823

Pt_4
4
SCF Energy =-17.5571627
Pt     0.17099    -0.96463    -1.30148
Pt     1.15094    -0.04848     0.83674
Pt    -1.23629    -0.22957     0.66084
Pt     0.13182    -2.35082     0.65798

Pt_4
4
SCF Energy =-17.6369645
Pt    -1.39844     0.08965    -0.63390
Pt     0.44957     0.71101     0.76332
Pt     0.77008    -0.85792    -1.02251
Pt    -1.00768    -1.25114    -2.58404

Pt_5
5
SCF Energy =-22.0659986
Pt    -1.48624     0.04655    -0.04025
Pt    -0.35093     0.23543     2.04709
Pt     0.84215    -0.40693     0.09512
Pt     2.08540    -0.84604    -1.88166
Pt     3.12053    -1.06192     0.25459

Pt_5
5
SCF Energy =-22.1409661
Pt    -0.77016    -0.60268    -0.40602
Pt     0.54698    -1.11909     1.65165
Pt     1.17923     0.67673     0.19816
Pt    -0.05500     1.21782    -1.79831
Pt     1.97262     2.50992    -1.12366

Pt_6
6
SCF Energy =-26.6690655
Pt    -0.12912    -1.57110    -1.28205
Pt     0.42546     0.19457     0.66652
Pt     1.72809    -1.68274     0.14351
Pt    -1.76636     0.51689    -0.85487
Pt    -1.11713     1.94600     0.88592
Pt    -2.08075    -1.12284    -2.50042

Al_3
3
SCF Energy =-2.8841358
Al    -0.40180    -1.17076    -0.49627
Al     0.33271     1.28780     0.10806
Al    -0.41942     0.70344    -2.35007

Al_4
4
SCF Energy =-3.8884019
Al    -1.08545    -0.67363    -0.48182
Al     0.22644     1.54688     0.17066
Al     1.35505    -0.72675     0.57503
Al     2.15058     1.37427     1.99948

Al_5
5
SCF Energy =-4.8889423
Al    -1.32360    -1.07341    -1.08105
Al    -0.37327     1.12796    -0.17882
Al     0.73778    -1.28143     0.59353
Al     1.61592     1.19405     1.45217
Al     2.77280    -0.93740     2.27789

Al_6
6
SCF Energy =-5.9187904
Al     0.88873    -1.27195     0.73110
Al     1.33084     1.41363     1.18836
Al     0.44373     0.65285    -1.19845
Al    -1.16095     0.57782     0.79049
Al    -1.16214    -1.43521    -0.92930
Al     2.80741    -0.38986     2.31219

Al_7
7
SCF Energy =-6.9567339
Al         0.19791       -1.47602        0.6711[illegible]
Al         0.96014        1.18861        1.0079[illegible]
Al        -0.07502        0.41952       -1.2996[illegible]
Al        -1.53852        0.59028        1.1859[illegible]
Al        -2.13364       -1.04586       -0.8026[illegible]
Al         2.33966       -0.39044       -0.5304[illegible]
Al        -2.26444       -1.92908        1.6397[illegible]

Al_8
8
SCF Energy =-7.9929798
Al     0.12238    -1.43567     0.52702
Al     1.34859     0.89966     0.74175
Al     0.35444     0.02087    -1.73970
Al    -1.45379     0.68809     0.17380
Al    -1.81250    -1.47992    -1.31446
```

```
Al     2.01855    -1.08035     2.34169
Al     0.04224     2.58805    -0.74421
Al     2.36169     1.72587    -1.60097

Al_3
3
SCF Energy =-727.047801031
Al    -0.15941    -0.36608    -1.14407
Al     0.56200     0.23999     1.29339
Al     2.22816    -1.24414    -0.59830

Al_4
4
SCF Energy =-969.431421176
Al     0.45669     1.22179     0.02858
Al     0.03145    -1.48327    -0.00097
Al     1.85323    -0.90542     1.61053
Al     2.27849     1.79943     1.64008

Al_5
5
SCF Energy =-1211.84013745
Al    -0.58707     0.79815    -1.64876
Al    -1.32183    -1.43499    -0.74232
Al     0.53533    -0.57307     0.87532
Al     0.55122     1.95990     0.57411
Al    -1.66377     2.79802    -0.60460

Al_6
6
SCF Energy =-1454.25169473
Al     1.02424     0.38687    -0.91293
Al     1.46992    -0.04909     1.61168
Al     0.12791    -2.04516    -0.70407
Al    -0.59503     1.45678     1.13063
Al    -1.72540    -0.22528    -0.82630
Al    -1.78738    -0.77857     1.71567

Al_7
7
SCF Energy =-1696.68138575
Al     1.02391    -1.18280     0.30699
Al     1.01991     0.15816     2.52482
Al    -1.30396    -0.97410    -0.96347
Al    -1.28602     0.33056     1.35617
Al     0.15500     1.25052    -1.05503
Al     2.34871     1.05043     0.32129
Al     0.19980     2.44849     1.24309

Al_8
8
SCF Energy =-1939.07923013
Al    -1.33175     0.07330    -0.31865
Al     1.01003    -2.90221     1.04269
Al    -0.55977    -1.07274     1.91711
Al    -0.13228     1.43168     1.91210
Al    -0.32806     3.01600    -0.09125
```

```
Al     0.80301    -1.31373    -0.96021
Al     1.24499     1.18844    -0.96291
Al     2.00404     0.04553     1.27258

Al_3
3
SCF Energy =-2.8841358
Al    -0.40180    -1.17076    -0.49627
Al     0.33271     1.28780     0.10806
Al    -0.41942     0.70344    -2.35007

Al_4
4
SCF Energy =-3.880284
Al    -1.32575     0.64859    -0.59852
Al     0.94766     0.92552     0.90469
Al     0.03328    -1.62429     0.10241
Al    -1.36192    -0.13355     1.92879

Al_5
5
SCF Energy =-4.8711797
Al    -0.96587    -1.22127    -0.42732
Al     0.19106    -0.37145     1.80225
Al     1.25747     0.22437    -0.55582
Al    -1.02307     1.35523     0.19710
Al    -2.45582    -0.38283     1.60408
```

RuPt– Binary Clusters:

```
Pt_1Ru_1
2
SCF Energy =-7.4735132
Pt    -0.60418    -0.11282     0.40247
Ru    -2.43861    -0.45535     1.62450

Pt_1Ru_3
4
SCF Energy =-13.7251589
Pt     0.44501     0.19961    -1.65996
Ru    -1.39135     0.48957     0.07289
Ru     0.77099     0.26719     0.93963
Ru    -1.32079     2.13687    -1.58940

Pt_4Ru_3
7
SCF Energy =-27.6281741
Pt     1.76410    -0.97430    -0.68192
Ru    -0.02455     0.18625     0.19244
Pt    -1.12067     1.97867    -1.03070
Pt     1.24522     1.63002     1.75544
Pt    -1.50829    -0.80346     1.84268
Ru    -1.14087    -1.16215    -1.27059
Ru    -1.25302     1.78707     1.57498

Pt_2Ru_5
7
```

```
SCF Energy =-24.591675
Pt     0.67927    -1.66647     0.41144
Ru    -1.33490    -0.37215     0.35299
Pt    -1.64544     1.97869     0.68677
Ru     0.28653     0.74331    -1.09400
Ru     1.52940    -0.98621    -1.97689
Ru     1.31457     1.30446     0.89553
Ru    -0.82696     2.66490    -1.70824

Pt_2Ru_4
6
SCF Energy =-21.4773971
Pt     1.43870    -1.23800     0.66186
Ru    -1.06575    -0.49269     1.35585
Pt    -1.49256     2.05164     0.77358
Ru     0.27479     0.75244    -0.20560
Ru     1.29582    -0.34604    -1.93630
Ru     1.38626     2.11305     1.29140

Pt_2Ru_2
4
SCF Energy =-15.2286277
Pt     1.46056    -1.70702    -0.65726
Ru    -0.15731    -0.10959     0.61987
Pt    -1.75496     0.99398    -1.12114
Ru    -0.14014    -0.52251    -1.90745

Pt_3Ru_3
6
SCF Energy =-22.9939335
Pt     0.03954    -3.01366    -0.04326
Ru     0.00361    -0.66442    -0.20470
Pt    -1.50595     0.40002    -1.96031
Pt     1.34377     0.75384     1.25007
Ru    -0.51155     1.71951    -0.06052
Ru    -0.70829    -1.97930    -2.25339

Pt_4Ru_2
6
SCF Energy =-24.477204
Pt     1.28397    -1.49612    -1.27155
Ru    -0.30016    -0.16365    -0.23710
Pt    -1.35505     1.33935    -1.64856
Pt     0.89940     1.14326     1.17032
Pt    -1.74423    -1.32669     1.14860
Ru    -1.56930    -1.53070    -1.72926

Pt_2Ru_3
5
SCF Energy =-18.3596244
Pt     1.49028    -1.39676    -0.00281
Ru    -1.05216    -1.25506     0.27892
Pt    -1.54904     1.12047    -0.48932
Ru     0.94505     0.91829    -0.95545
Ru    -0.29346    -0.87284    -1.96681

Pt_2Ru_1
3
SCF Energy =-12.0500323
Pt     1.01026     0.07702    -0.58652
Ru    -1.08779     0.45458     0.21511
Pt    -1.66752     2.33232    -0.93729

Pt_3Ru_1
4
SCF Energy =-16.6559347
Pt     0.88629    -0.60882    -0.43380
Ru    -0.63678    -0.19976     1.43086
Pt    -0.42720     1.56071    -0.41694
Pt    -1.96229     1.84551     1.58153

Pt_3Ru_4
7
SCF Energy =-26.113489
Pt     1.46817    -2.54008     0.24339
Ru     0.39782    -0.23584     0.32941
Pt    -1.42464     0.81054    -0.95835
Pt     2.09305     1.11262     1.74394
Ru     0.09576     2.11982     0.62476
Ru    -0.63559    -1.80388    -1.07794
Ru     1.15899    -1.20142     2.41800

Pt_3Ru_2
5
SCF Energy =-19.8352831
Pt     0.78107    -2.23399    -0.31263
Ru     0.22301    -0.08079    -0.18314
Pt    -1.07973     1.08127    -2.04241
Pt     1.47734     1.54080     1.33530
Ru     0.23134     2.27219    -0.50870

Pt_6Ru_1
7
SCF Energy =-30.5454408
Pt     2.11940    -0.91355    -1.20381
Ru    -0.10106    -0.35724    -0.49480
Pt    -0.90984     1.05608    -2.08849
Pt     1.53848     0.91355     0.70627
Pt    -1.95551    -1.69898     0.21606
Pt    -1.03122     0.48366     1.54814
Pt    -0.07892    -2.49316    -1.58281

Pt_1Ru_6
7
SCF Energy =-23.0763827
Pt     0.18988    -0.85727    -1.47329
Ru    -0.21279    -1.55960     0.82247
Ru     1.39803     0.00527     1.44609
Ru    -1.12157     0.71760     1.04413
Ru     0.69517     1.32622    -0.31026
Ru    -2.13347    -0.11802    -0.80556
Ru    -1.65151    -1.06365     2.54980

Pt_5Ru_1
```

```
6
SCF Energy =-25.9237115
Pt      1.42514     -0.93096     -0.33298
Ru     -0.46820      0.20300      0.26829
Pt     -1.28463      1.12286     -1.63157
Pt      1.06468      1.63409      1.39963
Pt     -1.75048     -1.59878      0.81176
Pt     -1.41393      1.38381      2.09580

Pt_4Ru_1
5
SCF Energy =-21.2667319
Pt      2.01124     -1.13595     -0.95482
Ru      0.21221      0.25227     -0.00215
Pt     -1.28284      1.54993     -1.47483
Pt      0.07341      0.53182      2.20093
Pt      0.66779      0.34661     -2.42157

Pt_1Ru_5
6
SCF Energy =-19.953578
Pt      0.84156     -0.76025     -1.33992
Ru     -0.76262     -1.66141      0.36334
Ru      0.82859      0.29771      1.10017
Ru     -1.54458      0.52895      0.74029
Ru     -0.25155      1.46696     -0.99011
Ru     -3.68872      0.57591      0.03866

Pt_5Ru_2
7
SCF Energy =-29.1059105
Pt      1.49819     -0.96298     -0.90933
Ru     -0.52524     -0.02780     -0.05716
Pt     -1.06806      0.92873     -2.12137
Pt      1.45983      0.78091      1.01265
Pt     -1.31001     -2.10777      0.66721
Pt     -1.34767      1.51817      1.54507
Ru     -2.90355      0.02592     -0.20993

Pt_1Ru_4
5
SCF Energy =-16.8396991
Pt      0.86247     -0.25646     -1.63499
Ru     -1.32810     -1.05767     -0.43507
Ru      1.17513      0.72264      0.78881
Ru     -1.08866      1.10131      0.40099
Ru     -0.62457      1.91213     -1.73062

Pt_1Ru_2
3
SCF Energy =-10.6241488
Pt      1.22787      0.35463     -0.16478
Ru     -0.91259     -0.13123      0.81207
Ru      1.67070      0.93312      2.12370
```

PtAu— Binary Clusters:

```
Pt_4Au_2
6
SCF Energy =-25.9716853
Au     -1.09970      1.95259     -0.79810
Pt     -0.66326     -0.53079     -0.18759
Au     -1.57232     -2.90095     -0.72102
Pt      0.81461      1.08420      0.87663
Pt      1.96916     -0.75041      2.01179
Pt      0.47484     -2.36287      0.93879

Pt_3Au_3
6
SCF Energy =-25.2928965
Au     -0.48417     -2.86768      1.79431
Pt     -0.37962     -0.85844      0.14485
Au      2.77385      0.88036      0.85052
Au     -1.52300      0.46690     -1.77989
Pt      0.77433      1.11734     -0.74363
Pt      1.48479     -1.16689      1.71135

Pt_2Au_1
3
SCF Energy =-12.8539196
Au     -0.50627     -0.23918      1.22873
Pt      1.04003      0.54752     -0.73989
Pt      1.76026      1.01781      1.64160

Pt_3Au_4
7
SCF Energy =-29.2368334
Au     -0.31726     -1.81541      1.52037
Pt      0.04636      0.49432      0.46633
Au      2.76633      0.58638     -0.16033
Au     -0.73947      1.05999     -2.15555
Au     -1.08176      2.29681      1.89832
Pt     -0.07049     -1.27425     -1.11561
Pt      1.14640      2.59849      0.39716

Pt_3Au_2
5
SCF Energy =-21.359583
Au     -2.42025     -0.96373      0.03480
Pt      1.38167     -0.68271     -2.21009
Au      0.92365      1.83234      1.09428
Pt      0.00758      0.09445     -0.35294
Pt     -0.81051     -1.72194     -1.96005

Pt_1Au_4
5
SCF Energy =-20.0475058
Au     -2.01554     -1.34457     -0.14298
Pt     -0.49146      0.70328     -0.17431
Au      1.56536      2.03895     -0.20645
Au      0.62639     -1.59251     -0.16373
Au     -2.27180      2.38961     -0.17294

Pt_2Au_5
```

```
7
SCF Energy =-28.5385619
Au    -1.40261    -1.42775     1.00908
Pt    -0.01234     0.44580    -0.42030
Au     0.00714     3.00253     0.42322
Au    -0.10890    -2.11506    -1.13991
Au    -1.37296     1.05115     1.78726
Au     1.33854    -0.30589    -2.62240
Pt     1.25751     2.22756    -1.79900

Pt_3Au_1
4
SCF Energy =-17.4244152
Au    -0.86046    -1.00895     0.44699
Pt     0.01416     0.72331    -1.44559
Pt     0.70437     1.15241     0.92284
Pt     1.44767     2.67760    -0.89507

Pt_24Au_3
5
SCF Energy =-20.6934629
Au    -0.63120    -1.61096     1.22137
Pt     0.10306     0.21055    -0.18160
Au     2.23329     1.55389     0.57904
Au    -1.29117     0.69915    -2.35737
Pt     0.83070     2.02460    -1.58144

Pt_4Au_1
5
SCF Energy =-22.0254682
Au    -1.01184    -1.93287     0.61634
Pt     0.04912     0.09049    -0.72536
Pt    -0.39501     0.42694     1.65013
Pt     0.93756     2.40224    -0.89128
Pt     0.49238     2.73918     1.49543

Pt_1Au_3
4
SCF Energy =-16.1352867
Au    -1.72285    -1.93617     0.34562
Pt     0.37911    -0.51163    -0.49380
Au     1.44053     1.60108    -0.02576
Au    -0.15728    -2.86550    -1.56551

Pt_1Au_6
7
SCF Energy =-27.879473
Au    -2.09201    -1.17485     0.88538
Pt     0.11608    -0.12703    -0.16387
Au     1.05157     2.26526     0.52666
Au    -0.66617    -2.47506    -0.94394
Au    -1.21384     1.27786     1.65356
Au     2.28575     0.75517    -1.28087
Au    -2.50960     2.64477     3.42251

Pt_4Au_3
7
SCF Energy =-29.9093563
Au    -2.49139    -0.47472     1.56071
Pt     1.52872     0.14028    -0.62394
Au     1.59583     2.47058     0.50756
Au     0.92762    -1.98705    -1.97622
Pt    -0.35524     0.97336     1.32492
Pt    -0.72343    -1.48268    -0.04367
Pt     1.22415    -1.09376     1.67235

Pt_6Au_1
7
SCF Energy =-31.2531691
Au    -1.86793    -1.21154     1.78539
Pt     0.69736    -1.08455    -2.65326
Pt    -0.64038    -1.27642    -0.54180
Pt     1.17166     0.49917    -0.87351
Pt    -0.01177     0.56075     1.38026
Pt     1.79873     2.24018     0.90034
Pt     2.94364     2.12543    -1.38215

Pt_1Au_2
3
SCF Energy =-12.2241319
Au    -0.69918    -0.34808     0.89921
Pt     0.94630     0.73271    -0.59262
Au     2.02385     2.44395     0.82559

Pt_5Au_2
7
SCF Energy =-30.586268
Au    -1.05682     2.60134    -1.07037
Pt    -0.81345     0.08740    -0.48275
Au    -1.52543    -2.41945    -0.99179
Pt     0.72044     1.68497     0.64342
Pt     1.88615    -0.16808     1.97722
Pt     0.27917    -1.80712     0.77913
Pt    -0.55407    -0.28281     2.67578

Pt_2Au_2
4
SCF Energy =-16.7835435
Au    -2.25663    -1.11283     0.11148
Pt     0.32459    -0.21891    -0.34635
Au     1.14902     2.18881    -0.02251
Pt    -1.43863     1.29652     0.44791

Pt_5Au_1
6
SCF Energy =-26.6459402
Au    -1.52740    -2.38746     0.05013
Pt     0.19535    -0.83776    -1.11926
Pt    -0.25773    -0.49749     1.30049
Pt     1.85720     0.69545    -2.09786
Pt     1.01971     1.32475     2.35858
Pt     1.94279     1.50553     0.15512

Pt_2Au_4
```

6			
SCF Energy =-24.621332			
Au	-1.56797	-1.56500	0.35333
Pt	0.04399	0.28020	-0.46155
Au	2.42825	1.19250	-0.94893
Au	1.22978	-1.86150	0.34863
Au	-2.19315	1.55212	-0.90397
Pt	0.20613	2.52382	-1.41488

6			
SCF Energy =-23.9781012			
Au	-1.39035	-1.13168	0.38978
Pt	0.73381	-0.02569	-0.56887
Au	1.73311	2.27626	0.02788
Au	0.31792	-2.30123	-1.59077
Au	-0.58566	1.37854	1.25594
Au	2.84061	0.44723	-1.88621

Pt_1Au_5
